# Structure-function analysis of ceTIR-1/hSARM1 explains the lack of Wallerian axonal degeneration in *C*. *elegans*

**DOI:** 10.1016/j.celrep.2023.113026

**Published:** 2023-08-26

**Authors:** Tami Khazma, Atira Grossman, Julia Guez-Haddad, Chengye Feng, Hadas Dabas, Radhika Sain, Michal Weitman, Ran Zalk, Michail N. Isupov, Marc Hammarlund, Michael Hons, Yarden Opatowsky

**Affiliations:** 1 The Mina & Everard Goodman Faculty of Life Sciences, Bar-Ilan University, Ramat-Gan, Israel; 2 Departments of Neuroscience and Genetics, Yale School of Medicine, New Haven, CT, USA; 3 Department of Chemistry, Bar-Ilan University, Ramat Gan, Israel; 4 Ilse Katz Institute for Nanoscale Science & Technology, Ben-Gurion University of the Negev, Beer-Sheva, Israel; 5 Henry Wellcome Building for Biocatalysis, Biosciences, Faculty of Health and Life Sciences, University of Exeter, Exeter, UK; 6 European Molecular Biology Laboratory, Grenoble, France; 7 These authors contributed equally; 8 Lead contact

## Abstract

Wallerian axonal degeneration (WD) does not occur in the nematode *C*. *elegans*, in contrast to other model animals. However, WD depends on the NADase activity of SARM1, a protein that is also expressed in *C*. *elegans* (ceSARM/ceTIR-1). We hypothesized that differences in SARM between species might exist and account for the divergence in WD. We first show that expression of the human (h)SARM1, but not ceTIR-1, in *C*. *elegans* neurons is sufficient to confer axon degeneration after nerve injury. Next, we determined the cryoelectron microscopy structure of ceTIR-1 and found that, unlike hSARM1, which exists as an auto-inhibited ring octamer, ceTIR-1 forms a readily active 9-mer. Enzymatically, the NADase activity of ceTIR-1 is substantially weaker (10-fold higher *K*m) than that of hSARM1, and even when fully active, it falls short of consuming all cellular NAD^+^. Our experiments provide insight into the molecular mechanisms and evolution of SARM orthologs and WD across species.

## In brief

Why do axons degenerate after injury in most model animals but not in *C*. *elegans*? Khazma et al. found that SARM1, which executes axon degeneration by depleting NAD^+^, is not nearly as active in *C*. *elegans*. Indeed, insertion of human SARM1 into *C*. *elegans* axons leads to degeneration after injury.

## Graphical Abstract

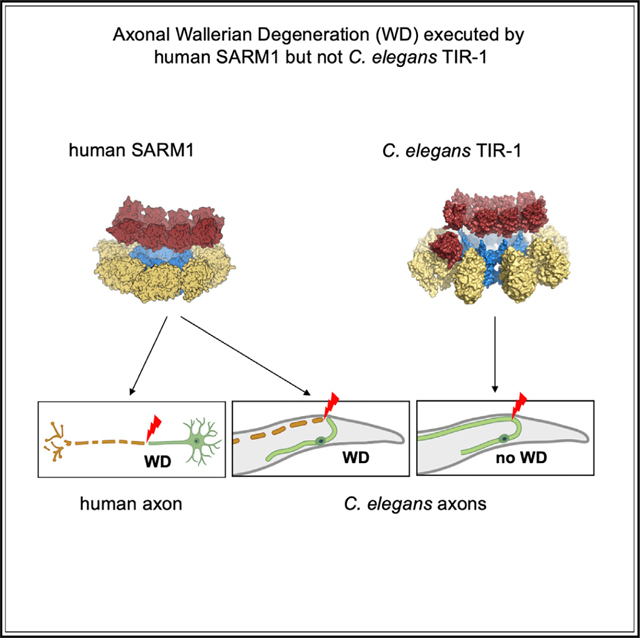

## INTRODUCTION

Following injury, the distal part of an axon undergoes a sequential degenerative process termed Wallerian degeneration (WD). WD was first described in frogs by the British physician Augustus Waller in the mid-19^th^ century,^1^ and it has been observed in a diverse group of animal models^2^ including *Drosophila*,^3,4^ zebrafish,^5,6^ and mouse.^7^ WD follows a morphological sequence that consists of a latent phase, followed by beading, thinning, fragmentation, and finally clearance of axonal debris.^8^ More recently, the molecular mechanisms that execute WD have been elucidated, where upon injury, the transport of the short-lived NAD-synthase NMNAT2 to axons is disturbed. This results in rapid degradation of NMNAT2, depletion of nicotinamide adenine dinucleotide (NAD^+^), and the accumulation of the NMNAT2 substrate nicotinamide mononucleotide (NMN).^9,10^ It was suggested that NMN elevation^11–13^ and/or NAD^+^ depletion^14,15^ activates the key pro-degenerative NADase SARM1 (sterile alpha and Toll/Interleukin-1 Receptor [TIR] motif containing 1),^16–18^ which prior to axon injury is kept inhibited and does not exert robust NADase activity.^19^ The essential role for SARM1 in promoting WD has been demonstrated in knockout studies across different species and contexts. For example, SARM1 NADase loss of function by genetic^20,21^ or pharmacological^22–26^ means inhibits WD and even rescues the development and survival of axons deficient for NMNAT2,^27^ while SARM1 gain of function induces WD.^28^

**Figure 1.**
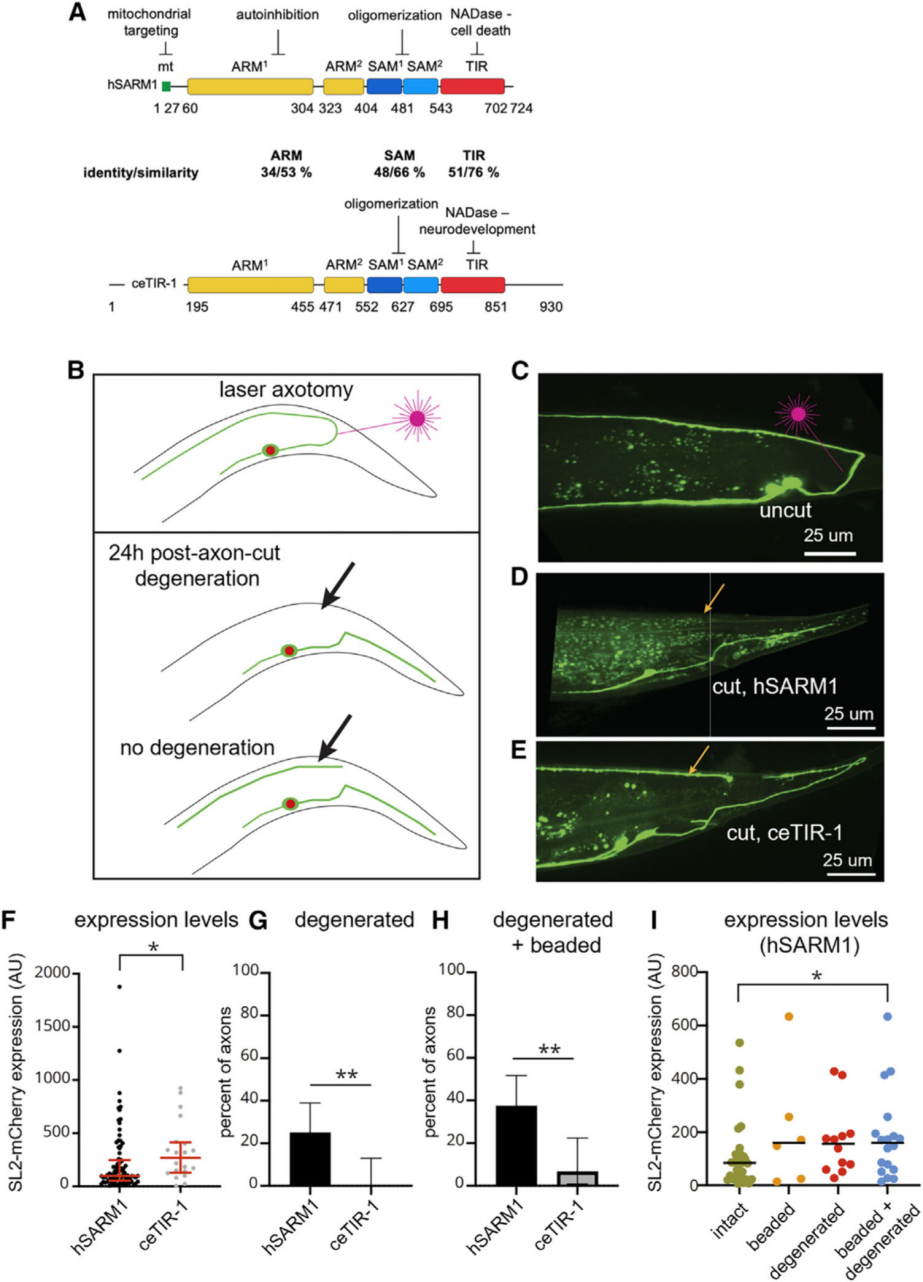
hSARM1, but not ceTIR-1, induces WD in *C*. *elegans* (A) Color-coded organization, nomenclature, and boundaries of the hSARM1 and ceTIR-1 domains (ARM, SAM, and TIR). (B) Scheme for DA9 neuron axotomy and analysis of degeneration. (C) Representative micrograph of animals prior to axotomy; site of typical laser axotomy is shown. (D) Representative micrograph of hSARM-overexpressing animal displaying degeneration 24 h after axotomy. Orange arrow indicates dorsal region where distal fragment is no longer present. (E) Representative micrograph of animal overexpressing ceTIR-1 displaying no degeneration 24 h after axotomy. Orange arrow indicates distal axon fragment. (F) Expression levels of transgene in hSARM and ceTIR-1 overexpressing lines. Each dot represents an animal. Median and interquartile range are shown in red. Significance was calculated by Kolmogorov-Smirnov test. (G) Quantification of axon degeneration. Bars indicate percent degenerated axons; error bars indicate 95% confidence interval, calculated by the modified Wald method. Significance was calculated by Fisher’s exact test. (H) Quantification of axon degeneration and beading. Bars indicate percent degenerated or beaded axons; error bars indicate 95% confidence interval, calculated by the modified Wald method. Significance was calculated by Fisher’s exact test. (I) Expression levels of transgene in hSARM animals, organized by outcome after axotomy. Each dot represents an animal. Bars represent medians. Significance was calculated by Kolmogorov-Smirnov test.

SARM1 was first described in *C*. *elegans*, where it is encoded by the *tir-1* gene^29^ and is referred to hereafter as ceTIR-1. ceTIR-1 and human (h)SARM1 have a conserved domain topology with an N-terminal ARM domain, followed by two SAM and one TIR domain (Figures 1A and S1). NADase activity was demonstrated for the TIR domains of both ceTIR-1^30–32^ and hSARM1.^28,33^ However, endogenous ceTIR-1 was shown to act by promoting MAP kinase signaling, both during neuronal development^29^ and in innate immunity.^34^ Further, *C*. *elegans* does not exhibit robust axonal degeneration after injury, and the limited degeneration that does occur is independent of ceTIR-1.^35,36^ Even in a context in which degeneration of a short axon segment is triggered by multiple injuries, ceTIR-1 is largely dispensable for degeneration.^37^ Finally, in a context in which axon degeneration is triggered by lack of axonal mitochondria, rather than nerve injury, ceTIR-1 and MAP kinase signaling suppress degeneration, rather than promoting it.^38^

These data raise the possibility that ceTIR-1 is mechanistically distinct from its counterparts in other species, and that differences in SARM function between *C*. *elegans* and other species might account for the differences in the axonal injury response.

We first compared the ability of human and *C*. *elegans* SARM to promote degeneration *in vivo*, and we found that expression of hSARM1 in *C*. *elegans* neurons confers robust axon degeneration in response to nerve injury. To understand the basis for the functional discrepancy between ceTIR-1 and hSARM1, we conducted structural and enzymatic investigations, comparing the human and *C*. *elegans* orthologs. We find that a series of interlinked molecular alterations in SARM kinetic properties, the order and strength of oligomerization, auto-inhibition, and regulation by nicotine nucleotides set the two orthologs functionally apart. Our results shed new light on SARM toxicity and the molecular evolution of axonal degeneration and demonstrate that interspecies differences in SARM function account for the lack of injury-induced WD in *C*. *elegans*.

## RESULTS

### Axonal degeneration in *C*. *elegans* can be executed by hSARM1 but not ceTIR-1

To test the idea that different activities *in vivo* of SARM orthologs account for the differential response to axon injury between *C*. *elegans* and other species, we overexpressed either hSARM1 or ceTIR-1 in the DA9 neuron of *C*. *elegans* and performed laser axotomy. We codon-optimized hSARM1 to ensure robust translation. To determine expression levels of the hSARM1 and ceTIR-1 transgenes, the plasmids also contained an SL2-spliced nuclear marker (H2B-mCherry). Co-transcription of this marker allows quantitative measurement of SARM transgene expression in DA9. Finally, strains also expressed myristoylated GFP to allow accurate assessment of intact and damaged axons.

Previously, we found that after axotomy of the DA9 neuron at the point where the axon joins the dorsal nerve cord, the regenerating axon grows over and sometimes fuses with the distal fragment, making it difficult to assess degeneration.^39^ To enable assessment of degeneration without confounding regeneration, we developed an assay in which the DA9 axon is severed closer to the cell body, at the dorsal-ventral midline (Figures 1B and 1C). We found that this injury results in robust but mis-guided regeneration, in which the regenerating axon grows toward the posterior (Figure 1B). Although the axon can sometimes loop around and eventually extend in the anterior direction toward the distal fragment (e.g., Figure 1E), at 24 h after injury, there is clear separation between the distal fragment and the regenerating axon in ~90% of injured axons. We discarded the 10% of animals without clear separation and used the remaining animals to assess degeneration.

Consistent with previous results, we found that severed axons of DA9 in L4 animals degenerate very poorly, similar to other *C*. *elegans* neurons at this stage.^35,39^ In control animals 24 h after axotomy, the distal fragment was still present in all cases (23/23 animals). Next, we tested the effect of hSARM or ceTIR-1 overexpression. We selected transgenic lines with similar expression in DA9 as assessed by the SL2-spliced H2B-mCherry reporter, with ceTIR-1 having slightly higher expression (p = 0.02, Kolmogorov-Smirnov test) (Figure 1F). Strikingly, overexpression of hSARM1 enabled WD after axotomy in 25% of animals (12/48). In these animals, the distal fragment was completely absent 24 h after axotomy (Figures 1D and 1G). By contrast, animals that overexpressed ceTIR-1 never exhibited robust degeneration (0/30 animals, p = 0.002 compared to hSARM, Fisher’s exact test) (Figures 1E and 1G). We also observed that some axon fragments in both hSARM (6/48) and ceTIR-1 (2/30) animals displayed obvious beading, likely a result of incomplete degeneration. Taken together, 37.5% of hSARM1 animals exhibited some form of degeneration after injury (18/48), compared to 6.7% of ceTIR-1 animals (2/30 animals, p = 0.003 compared to hSARM, Fisher’s exact test) (Figure 1H).

We never observed axon degeneration in the absence of axotomy in either the hSARM or ceTIR-1 overexpressing strains (n > 100 animals), indicating that the pro-degeneration function conferred by hSARM overexpression requires an initiating injury—consistent with SARM function in other species.^2–7^ Next, we investigated whether the hSARM1-mediated degeneration that we observed was associated with particularly high expression of hSARM1, perhaps explaining why hSARM1 expression does not confer degeneration in all cases. We compared expression levels of hSARM1, binned by each of the three observed outcomes of axon injury: degeneration, beading, or intact fragment (Figure 1I). Although there was no significant difference in hSARM1 expression between intact axons and either degenerated or beaded axons (intact vs. degenerated: p = 0.16; intact vs. beaded: p = 0.16; Kolmogorov-Smirnov tests), when we pooled degenerated and beaded axons, we did find a significant increase in overall expression (intact vs. degenerated + beaded: p = 0.04; Kolmogorov-Smirnov test). Nevertheless, animals with relatively high and relatively low expression could be observed in all three outcomes. We conclude that higher hSARM1 expression favors degeneration, but that unknown factors other than expression levels also control the outcome of degeneration after nerve injury in hSARM1 animals.

Overall, our experiments indicate that overexpression of hSARM1 in *C*. *elegans* neurons is sufficient to confer injury-induced WD, while equivalent overexpression of ceTIR-1 has little effect.

### The *in vitro* oligomeric state of ceTIR-1

**Figure 2.**
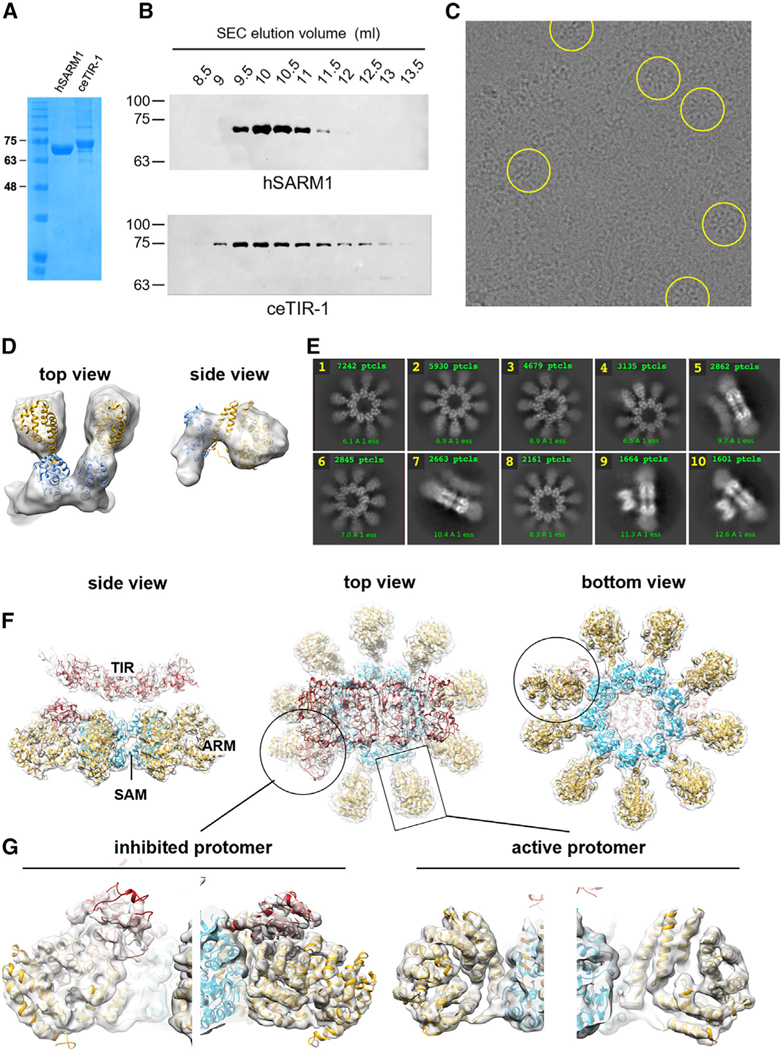
*In vitro* oligomerization and structure of ceTIR-1 (A) Ni-NTA purified hSARM1 and ceTIR-1 show similar levels in expression and purification in Coomassie stain SDS-PAGE. (B) Anti-FLAG WB gel filtration analysis demonstrates a much wider elution profile of ceTIR-1, compared with that of hSARM1 indicating for size heterogeneity of the former. (C) Exemplary cryo-EM micrograph of ceTIR-1, where clearly observed large ring-like particles are circled. (D) Cryo-EM 3D reconstruction of the small proteinaceous particles from the ceTIR-1 cryo-EM micrographs shows a low-resolution 3D volume of dimer where the prospective SAM domains mediate dimerization and are colored in light blue, while ARM domains extend outward and are colored in yellow. (E) Selected representation of 2D class averages from the large particles used for 3D reconstruction, indexed 1–10. The number of particles that were included in each average are indicated at the top of each class in green. Classes 1–4, 6, and 8 are face views, and classes 5, 7, 9, and 10 are side views. Note that while class 8 shows 10 protomers, the other face views have 9 protomers, which are more abundant. Asymmetry is observed in classes 2 and 4 where one of the peripheral spikes assumes a different conformation than the other eight. There are two types of side view presentations: classes 5 and 7 with a wide, arced perspective of the upper density (TIR domains array) and classes 9 and 10 with a narrow, tilted view of that density. (F) Color coded (as in Figure 1A) protein model docked in a transparent 3.5-Å cryo-EM density map (gray) reconstruction of 9-mer ceTIR-1. “Top view” refers to the aspect of the molecule showing the TIR (red) domains array closest to the viewer, while “side view” and “bottom view” are 90° and 180° tilts of “top view.” (G) Close side views of the asymmetric protomer, revealed as ARM-TIR complex in inhibited conformation, and of one of the eight active conformation ARM domains. A close view analysis of the TIR array is presented in Figure 5.

The different *in vivo* functions of hSARM1 and ceTIR-1 suggest that despite the overall conservation of these orthologs (Figure 1A), they vary in their molecular function. hSARM1 functions as a pre-formed homo-octameric ring, but the structure of ceTIR-1 is unknown. To determine the ceTIR-1 structure, a construct lacking the non-structural N- and C-terminal regions (^162^HMD … QQR^872^) was expressed and isolated. Expression levels and purity were similar to those of the hSARM1 that we have isolated and studied before^15^ (Figures 2A and 2B). However, we have noticed that the size exclusion chromatography elution peak of ceTIR-1 is distinctly broader than that of hSARM1 (Figure 2B), raising the possibility that ceTIR-1 is not as stably assembled as its human ortholog. Additionally, mass photometry analyses of purified hSARM1 and ceTIR-1 show a more uniform size distribution in hSARM1 than ceTIR-1 (Figure S3A). We have also noticed that cryoelectron microscopy (cryo-EM) micrographs of hSARM1^15^ and ceTIR-1 (this study Figure 2C) that were applied to the EM grids with similar concentrations show a remarkable lower density of ring-like particles in ceTIR-1. Finally, 3D reconstruction of small proteinaceous particles from ceTIR-1 cryo-EM micrographs yielded a low-resolution 3D volume of a dimer that well-accommodates two SAM-ARM modules (Figure 2D). We did not see similar smaller fragments in hSARM1 cryo-EM, and therefore, we suggest that the reason for this apparent difference is weaker electrostatic attraction forces between neighboring SAM^1−2^ domains in ceTIR-1, compared to hSARM1 (Figures S3B–S3D). These complementary electrostatic forces are the driving force of SARM1 oligomerization by holding the SAM^1−2^ inner scaffolding ring together.

In summary, we show that *in vitro*, ceTIR-1 is not as strongly assembled into ring oligomers as hSARM1 that are expressed and isolated under the same conditions. However, that does not necessarily reflect ceTIR-1 oligomerization state *in vivo*, which may be affected by the environmental conditions in *C*. *elegans* axons.

### The overall cryo-EM structure of ceTIR-1

To determine the 3D structure of ceTIR-1 (Figure 2C), we first performed particle picking and unsupervised 2D classification and averaging (Figure 2E). It revealed that, like hSARM1, ceTIR-1 is also assembled in a two-ring structure with an inner, scaffolding ring of SAM domains and a peripheral ring. However, unlike hSARM1, ceTIR-1 is mostly assembled into 9-mer (Figure 2E classes 1–4, 6) and, to a lesser extent, 10-mer (Figure 2E, class 8) rings. The peripheral ring clearly has a different conformation in ceTIR-1, where each ARM domain is separated from the neighboring ARMs and extends perpendicularly from the SAM domain’s inner ring. Notably, in the 9-mer rings (but not the 10-mer), one ARM domain adopts a tilted conformation and is dissimilar to the other eight protomers (Figure 2E, classes 2 and 4). Side views of the ring class averages reveal an additional density, with two distinct presentations, wide (Figure 2E, classes 5 and 7) and narrow (Figure 2E, classes 9 and 10), corresponding to TIR domain assemblies. Eventually, 121,487 particles were used in the 3D map reconstruction of 9-mer ceTIR-1. No symmetry constraints were applied, and a map with an overall 3.5-Å resolution was produced (Figure 2F, Table S1). AlphaFold^40^ atomic models of each domain were docked in the map density and were used as the starting model for further model building, adjustments, and refinement (Figure S5).

### The ceTIR-1 structure suggests constitutive activation

Besides consisting of nine subunits rather than eight, we observed several other key features of ceTIR-1 that suggest it functions differently from hSARM1. First, the TIR domains, the active part of the protein, are assembled differently. Eight of the nine TIR domains are packed in two slightly curved rows of four, with antiparallel 2-fold symmetry along the long and short axes. The placement of this array is not symmetrical in respect to the ring structure, rather tilted in both the long and short axes of the array. The ninth TIR domain is docked on one ARM domain that is positioned completely different than the other eight ARMs (Figures 2F, 2G, and S4A). A similar TIR domain array structure was recently discovered in NMN-activated hSARM1, which also requires NAD^+^ substrate (or better NAD-like orthosteric inhibitor, 1AD) to stabilize the TIR domain array.^25^ In stark contrast to that, the ceTIR-1 TIR array is readily assembled, without the need for NMN to release the TIR domains from ARM inhibition and without NAD^+^ involvement in stabilizing the TIR active array. Moreover, in the inhibited and active conformations of hSARM1,^15^ neighboring ARM domains are packed like roof tiles, where each one is partially covering the next (Figure S4A). In the ceTIR-1 ring 9-mer, eight ARM domains are arranged completely the other way around, with the amino terminal helix ARM^1^-α1 closest to the SAM domains, engaging in several close hydrophobic, polar, and electrostatic interactions with both SAM^1^ and SAM^2^ (Figures S4A and S4B). Sequence analysis of the interacting residues between the ceTIR-1 ARM^1^-α1 and SAM^1−2^ domains reveals a complete lack of conservation of these motifs beyond the phylum Nematoda (Figure S4C), and it is therefore unlikely that hSARM1 would adopt such a conformation.

In the inactive hSARM1, each TIR domain interacts with two neighboring ARM domains in a way that keeps the TIR domains apart from each other to prevent premature NADase activity. The ninth ARM domain of ceTIR-1 interacts with one TIR domain, adopting a similar conformation as in the inhibited hSARM1 (Figure S4A). In conclusion, at least *in vitro*, the portion of ceTIR-1 particles that do form ring oligomers are assembled in an active conformation with the TIR array pre-formed even without NAD^+^ and mostly free from ARM inhibition.

### ceTIR-1 has weak *in vitro* NADase activity compared to hSARM1

**Figure 3.**
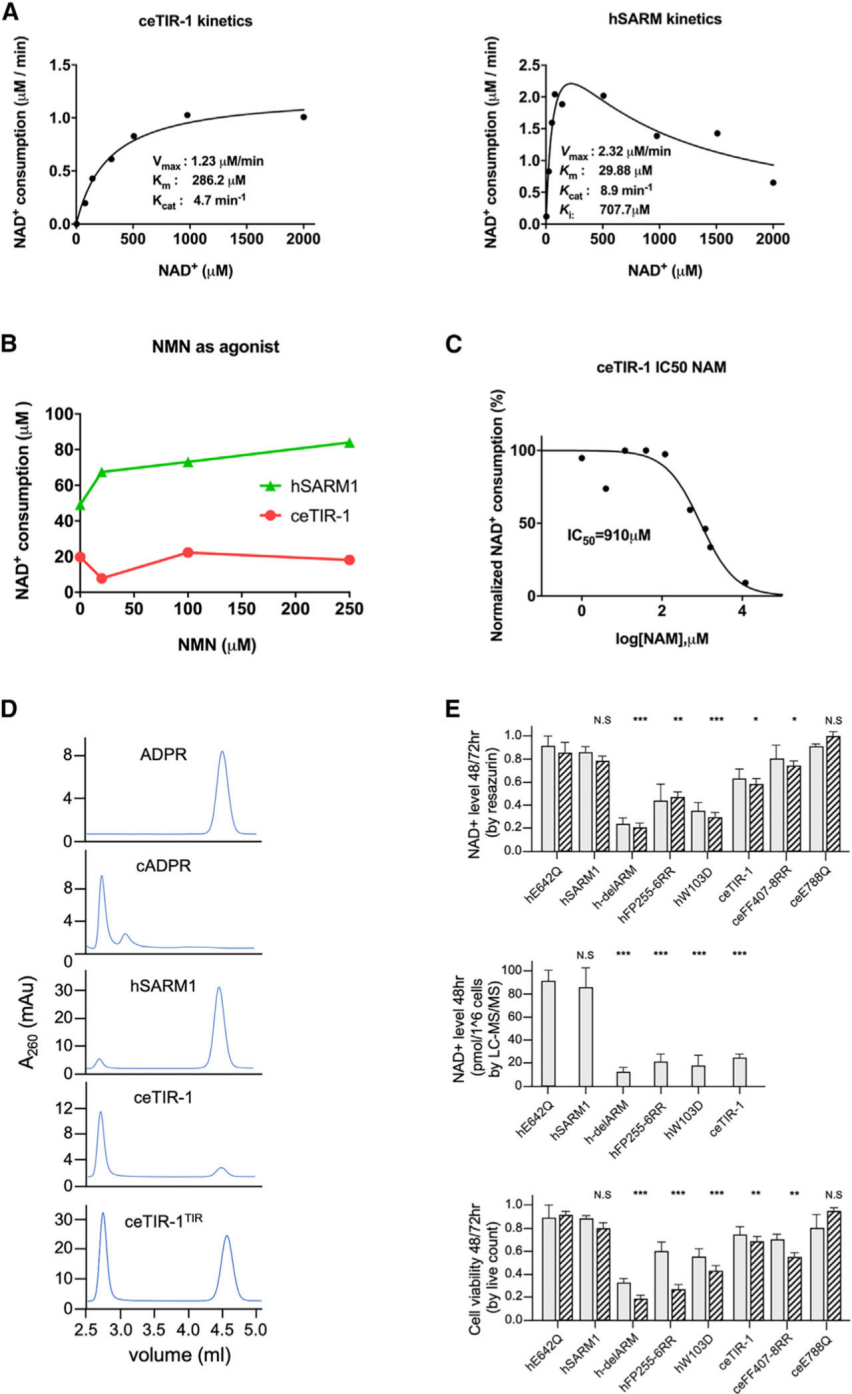
Enzymatic similarities and differences between hSARM1 and ceTIR-1 *in vitro* and in cell culture (A) Michaelis-Menten kinetics reveal a 103 higher *K*m of ceTIR-1 relative to hSARM1. Similar enzyme concentrations (260 nM) were incubated with varying NAD^+^ concentrations for 10 min (hSARM1) or 30 min (ceTIR-1). Inhibition by the NAD^+^ substrate is observed in hSARM1 but not ceTIR-1. Data points represent the mean of three measurements. The kinetic parameters were determined from plots of reaction velocity of NAD^+^ consumption versus substrate (NAD^+^) concentration and then fitted to the Michaelis-Menten equation (*K*m and *V*max) or substrate inhibition equation (*K*i) using non-linear curve fit in GraphPad Prism. *K*cat was calculated by dividing the *V*max with protein molar concentration. (B) NADase activity with nicotinamide mononucleotide (NMN) supplement. ceTIR-1 and hSARM1 were pre-incubated with different concentrations of NMN for 10 min at room temperature, before 150 mM NAD^+^ was added to each reaction. All the reactions were stopped after 30 min, and NAD^+^ levels were measured by HPLC. While hSARM1 responds to NMN with a 30%–50% elevation of activity, ceTIR-1 is unresponsive. Data points represent the mean of three measurements. (C) Nicotinamide (NAM) inhibits ceTIR-1 NADase activity. Various concentrations of NAM were pre-incubated with ceTIR-1 for 10 min at room temperature, followed by the addition of 150 mM NAD^+^, initiating the NADase activity. The assay was carried out at 27°C for 60 min. The amount of NAD^+^ consumption was measured by HPLC and used to calculate the IC_50_ values by plotting the normalized response to the dose of the NAM (log10). All calculations were performed in GraphPad Prism software. (D) C18-HPLC profiles of synthetic ADPR and cADPR (final concentration 50 mM) (upper two chromatograms) and HPLC NADase profiles of hSARM1, ceTIR-1, and ceTIR-1^TIR^ (lower three chromatograms). Similar amounts of hSARM1 and ceTIR-1 (260 nM) were incubated with 150 mM NAD^+^ for 120 min at 37°C and 27°C respectively. 74.78 mM ceTIR-1^TIR^ was incubated with 500 mM NAD^+^ for 30 min in 27°C. (E) Unlike hSARM1, the NADase activity of ceTIR-1 is not inhibited in cultured cells. NAD^+^ consumption in HEK293F cells was measured by the fluorescence resazurin assay (top) and by LC-MS/MS (middle). Cellular toxicity was measured by live count (bottom). ceTIR-1 activity in cultured cells is significant in both the resazurin and cell death assays but is the most conspicuous in the LC-MS/MS measurements, where NAD^+^ levels drop by 75% relative to NADase-negative control (hSARM1^E642Q^) or to hSARM1. Point mutations designed to disturb the ARM-TIR interaction ceFF407–8RR do not lead to further significant NAD^+^ consumption (resazurin) or cell death, demonstrating that ARM-TIR interactions do not play a significant role in ceTIR-1 inhibition. Gray and black-striped bars show values gained 48 and 72 h post transfection, respectively. Three biological repeats, Student’s t test; ***p < 0.001; **p < 0.01; *p ˂ 0.05; n.s., no significance.

To determine the intrinsic activity of ceTIR-1, we performed *in vitro* enzymatic studies. First, we determined the impact of temperature over the *in vitro* NADase activity of isolated ceTIR-1 and hSARM1. While hSARM1 has a narrow temperature preference at 37°C, ceTIR-1 is active at a wider range, from 22°C to 37°C (Figure S6A). Because the cultivation temperature of *C*. *elegans* usually does not exceed 25°C, we performed the *in vitro* ceTIR-1 measurements at this temperature and hSARM1 measurements at 37°C. We next measured the NADase activity of different concentrations of ceTIR-1 over time and found that the linear activity rate (steady state) extends to about 1 h, compared to 15 min for hSARM1 (Figure S6B). Michaelis-Menten kinetic measurements at similar enzyme concentrations (260 nM) showed that ceTIR-1 has about 10-fold higher *K*m and 2-fold lower *K*cat than hSARM1 (Figure 3A).

We also found that while hSARM1 is inhibited by high concentrations of the substrate NAD^+^ (substrate inhibition), ceTIR-1 is not (Figure 3A). Similarly, ceTIR-1 was not affected by nicotinamide mononucleotide (NMN), which elevates hSARM1 activity (Figure 3B).

NAM, a product of NADase activity (Figure S2), inhibited ceTIR-1 (Figure 3C) as was previously demonstrated for hSARM1.^20,24,26^ Notably, we found that the chemical structure of the reaction products (Figure 3D) is different between the two orthologs: hSARM1 generates about 90% ADPR and 10% cADPR, while ceTIR-1 yields nearly opposite ratios (Figure 3D). However, at high protein concentrations, the isolated TIR domain of ceTIR-1 (ceTIR-1^TIR^) (Figure S7A) can also produce substantial amounts of ADPR in addition to cADPR (Figures 3D and S7B).

The bias of ceTIR-1 toward cADPR enabled us to investigate the mechanism by which NAM inhibits SARM1. NAM is produced at the first stage of NAD^+^ catalysis, regardless of ADPR or cADPR production in the second stage (Figure S2). It is an *in vitro* inhibitor of SARM1, with an IC_50_ of 43–140 mM,^20,24,26^ and based on its measured levels in brain extracts (46 ± 13 mM),^41^ it is likely to contribute to SARM1 inhibition *in vivo*. However, it was recently suggested that what seems like competitive inhibition by NAM could actually be a result of a base-exchange reaction, in which NAM re-attaches to the newly formed ADPR-enzyme complex.^18^ We reasoned that such a base-exchange reaction would not affect the production of cADPR, in which cyclization replaces the need for an outside base (Figure S2). We found that increasing concentrations of NAM inhibit the production by ceTIR-1^TIR^ of both ADPR and cADPR alike (Figure S7C). Based on this result, we conclude that, to a large extent, inhibition by NAM is not achieved through base-exchange reaction, or else NAM would not have inhibited the production of cADPR.

### Unlike hSARM1, the NADase activity of ceTIR-1 is not suppressed in cell culture

To avoid premature activation, hSARM1 is kept inhibited *in vivo*, showing only residual NADase activity when compared to hSARM1 that bears activating mutations, such as those that compromise ARM domain inhibition (e.g., truncation of the entire ARM, hSARM1^delARM^; mutation of the ARM-TIR interface, hSARM1^FP255–6RR^; or mutation of the NAD^+^ allosteric inhibitory site, hSARM1^W103D^).^15^ We found that despite its weaker *in vitro* activity (Figure 3A), ceTIR-1 has substantially higher NADase activity than hSARM1 in cells (Figure 3E). In particular, direct measurements of NAD^+^ by LC-MS/MS found that cells expressing ceTIR-1 have only 25% of NAD^+^ relative to negative control (hSARM1^E642Q^) or to hSARM1. Lower NAD^+^ was also observed indirectly using resazurin, and cells expressing ceTIR-1 had reduced viability relative to control (hSARM1^E642Q^) or to hSARM1. Point mutations in ceTIR-1 designed to disturb the ARM-TIR interaction (ceFF407–8RR) did not lead to further significant NAD^+^ consumption (resazurin) or cell death, demonstrating that ARM-TIR interactions do not play a significant role in ceTIR-1 inhibition and suggesting that ceTIR-1 is already fully active when expressed in cultured cells.

Overall, our biochemical analysis is consistent with our structural data: compared to hSARM1, ceTIR-1 is not significantly inhibited in cells, but it has reduced NADase activity. Additional novel features are lack of regulation by NAD^+^ and NMN and the predominance of cADPR production over ADPR.

### Structure and properties of hARM-SAM-ceTIR CHIMERA

**Figure 4.**
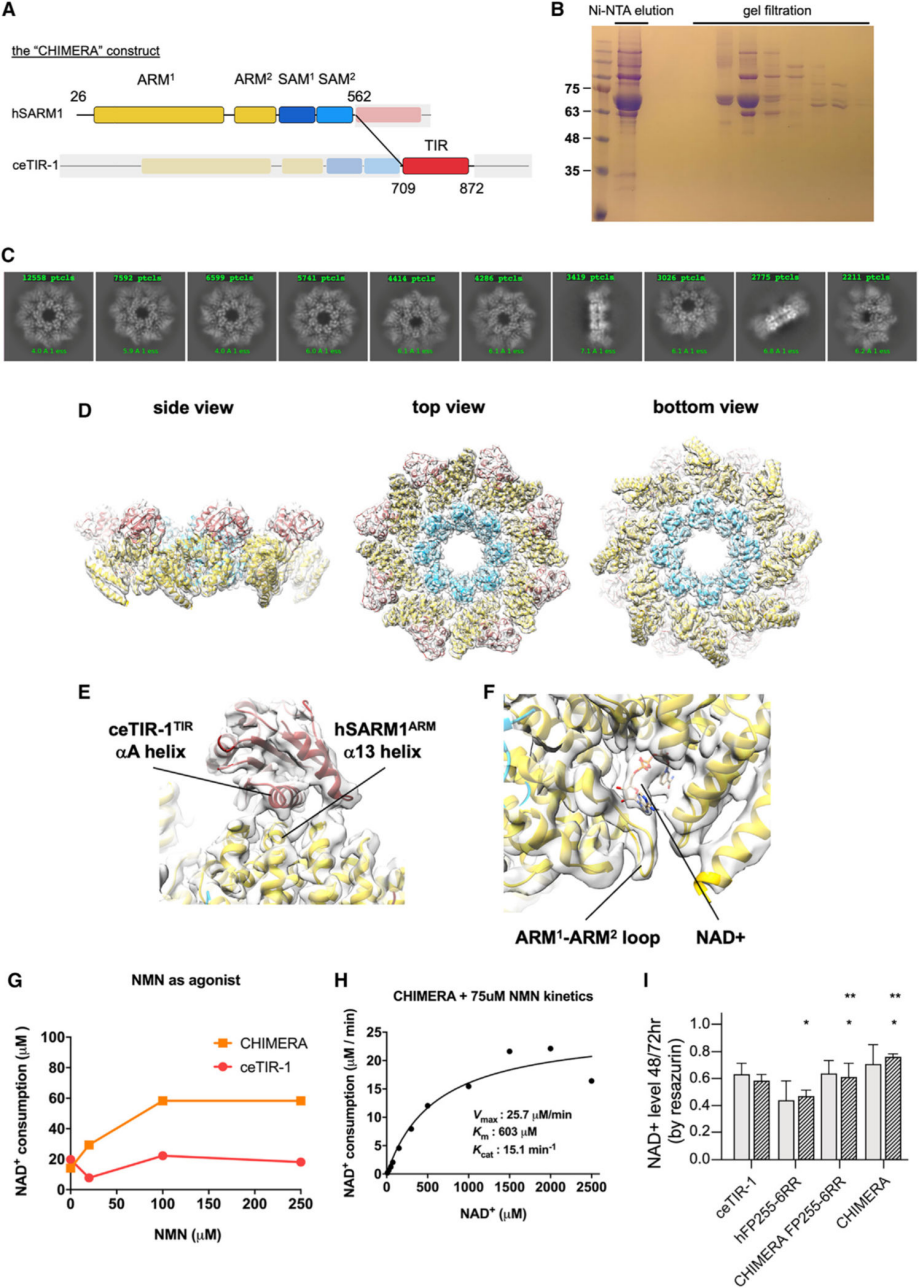
Structure and kinetics of CHIMERA (A) Diagram of the CHIMERA construct as a fusion of the ARM and SAM domains of hSARM1 and the TIR domain of ceTIR-1. (B) Coomassie-stained SDS-PAGE of purified CHIMERA. (C) Selected representation of 2D class averages used for the 3D reconstruction of CHIMERA. The number of particles included in each average is indicated at the top of each class. (D) Color coded (as in Figure 1A) protein model docked in a transparent 2.8-Å cryo-EM density map (gray) reconstruction of octamer CHIMERA supplemented with 5 mM NAD^+^. “Top view” refers to the aspect of the molecule showing the TIR (red) domains array closest to the viewer, while “side view” and “bottom view” are 90° and 180° tilts of “top view.” (E) Zoom-in view of the interacting ceTIR-1 TIR domain with the hSARM1 ARM. (F) Zoom-in view of the inhibitory allosteric NAD^+^ binding site at the concave surface of the hSARM1 ARM domain. In this structure, the ARM^1^-ARM^2^ loop assumes a more compact conformation than observed in other hSARM1 published structures. (G) Effect of NMN supplement over the NADase activity of CHIMERA. CHIMERA was pre-incubated with different concentrations of NMN for 10 min at room temperature, before 150 mM NAD^+^ was added to each reaction. All the reactions were stopped after 30 min, and NAD^+^ levels were measured by HPLC. Like hSARM1 (see Figure 3B), CHIMERA responds to NMN with a significant elevation of activity, while ceTIR-1 does not. Data points represent the mean of three measurements. (H) Kinetic measurements of CHIMERA. 1.3 mM of CHIMERA was pre-incubated with 75 mM of NMN for 10 min at room temperature before various concentrations of NAD^+^ were added. The assay was carried out at 37°C for 10 min. The kinetic parameters were determined from plots of reaction velocity of NAD^+^ consumption versus substrate (NAD^+^) concentration and then fitted to the Michaelis-Menten equation (*K*m and *V*max) using non-linear curve fit in GraphPad Prism. *K*cat was calculated by dividing the *V*max with protein molar concentration. Data points represent the mean of three measurements. (I) Unlike ceTIR-1, and like hSARM1, the NADase activity of CHIMERA is inhibited in cultured cells. Point mutations designed to disturb the ARM-TIR interaction CHIMERA^FP255–6RR^ increase NAD^+^ consumption (resazurin), but not as strongly as hSARM1^FP255–6RR^, further demonstrating the weaker NADase activity of the ceTIR-1 TIR domains compared to those of hSARM1. Gray and black-striped bars show values gained 48 and 72 h post-transfection, respectively. Three biological repeats, Student’s t test; ***p < 0.001; **p < 0.01; *p ˂ 0.05; n.s., no significance.

Unlike hSARM1 that forms stable octamers, we found that the oligomeric state of purified ceTIR-1 is not fixed (Figures 2B–2D and S3A). Such unstable oligomerization might bias kinetic measurements of NADase activity, which requires oligomerization. To assess the activity of assembled *C*. *elegans* TIR domains on a stable SAM scaffold, we designed a chimeric protein with human ARM and SAM domains and a *C*. *elegans* TIR (h^26^ERL … DTP^562^-ce^709^DVF … QQR^872^), simply designated henceforth as “CHIMERA” (Figure 4A). CHIMERA was expressed and purified in the same way as hSARM1 and ceTIR-1, with similar yields (Figure 4B). Cryo-EM 2D classification (Figure 4C) and 3D reconstruction (Figure 4D) of CHIMERA supplemented with 5 mM NAD^+^ revealed structure similar to that of intact hSARM1 (e.g., PDB: 7ANW; EMD: 11834). The structure was refined to 2.7-Å resolution (Table S1) and allowed a better resolution of the ceTIR-1 TIR domain than that reconstructed in the intact ceTIR-1 structure. Apparently, the high level of sequence similarity between ceTIR-1 and hSARM1 at the TIR interacting interface with ARM (Figure S1) allows the *C*. *elegans* TIR to fit and dock well with the hSARM1 ARM (Figure 4E). Like hSARM1, CHIMERA showed a significant increase in NADase activity in response to NMN supplementation (Figure 4G), indicating for a TIR release from ARM inhibition following NMN binding to the ARM allosteric site. Substrate inhibition by high NAD^+^ concentration, which is another hSARM1 hallmark, was less prominent in CHIMERA (Figure 4H). NADase kinetic measurements of CHIMERA found that it has a 3-fold higher *K*cat than ceTIR-1. This is consistent with the better SAM oligomerization scaffold in CHIMERA that elevates the local concentration of enzymatically active TIR sites. On the other hand, the *K*m value of CHIMERA is even higher than that of ceTIR-1 (which by itself is about 10-fold higher than that of hSARM1), affirming that the *K*m is determined by intrinsic TIR domain properties. We next designed and expressed in HEK293F cells a CHIMERA bearing the ARM domain activating mutation FP255–6RR. This mutation disturbs the ARM-TIR interface and when introduced in hSARM1 (hSARM1^FP255–6RR^) inflicts strong elevation in both NADase activity and cell death, as we have demonstrated here (Figure 3E) and before.^15^ We found that the cellular NADase activities of CHIMERA and CHIMERA^FP255–6RR^ are weaker than hSARM1^FP255–6RR^ and even weaker than ceTIR-1 (Figure 4I).

In conclusion, purified ceTIR-1 is not fully oligomerized and therefore not fully NADase active. Nevertheless, forced oligomerization of the ceTIR-1 TIR domains in CHIMERA and the constitutively active mutant CHIMERA^FP255–6RR^ demonstrate that even when fully oligomerized and not inhibited, the NADase activity of the ceTIR-1 TIR domains is intrinsically weaker than that of hSARM1 both *in vitro* and in cell culture.

### Structural analysis of the ceTIR-1 TIR active array

**Figure 5.**
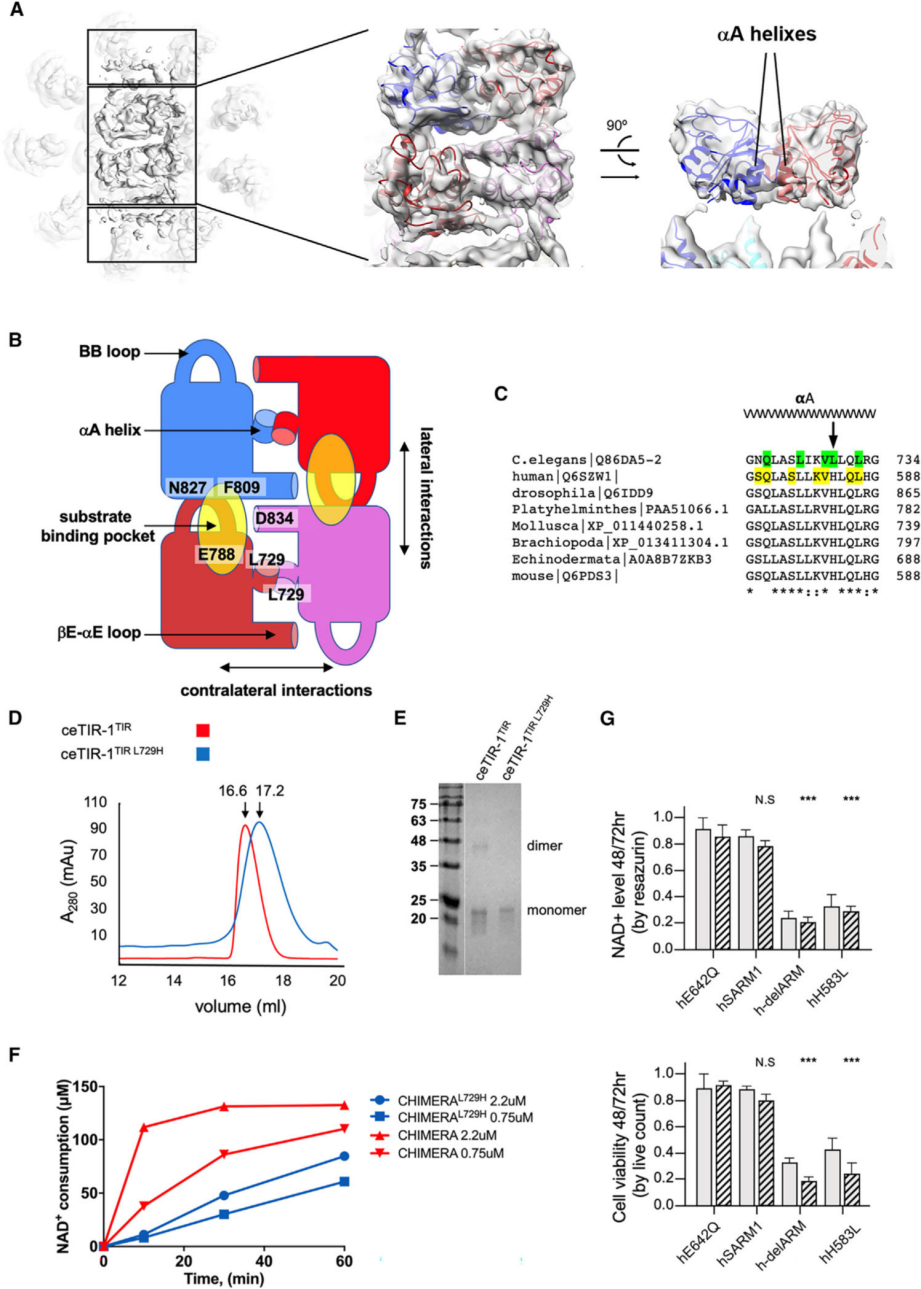
Assembly of the ceTIR-1 TIR domain active array (A) A top view of protein model docked in a high-contour transparent 3.5-Å cryo-EM density map (gray) of the 9-mer ceTIR-1. Note that the four central TIR domains have stronger map density than the peripheral ones. (B) An illustration of the four central TIR domains highlighting key elements in the TIR domain active array assembly and in NAD^+^ binding and catalysis. The array is constructed via two types of TIR-TIR interactions: counter-lateral, which are chiefly mediated by homotypic interactions of the TIR αA helices; and lateral interactions that involve the BB loop of one protomer and the back side of the next one (particularly F809). The lateral contacts are critical for NADase activity because only by that the BB loop opens and the NAD^+^ binding site is formed. (C) Multiple sequence alignment of the TIR domain’s αA helix. Highlighted in yellow are the residues that are directly engaged in the TIR domain inhibitory interaction with the ARM domain, and in green are those that are directly engaged in the activating homotypic TIR-TIR interactions. Note that the nematode ceTIR-1 L729 is the only animal that diverges from the otherwise conserved histidine in that position. (D) Gel filtration analysis shows an earlier elution of ceTIR-1^TIR^ than ceTIR-1^TIR L729H^, indicating for a smaller oligomeric state of the latter. (E) Glutaraldehyde crosslinking demonstrates a dimeric assembly of ceTIR-1^TIR^ but only monomeric of ceTIR-1^TIR L729H^. (F) *In vitro* NADase activity of purified CHIMERA is diminished by the L729H mutation. Data points represent the mean of three measurements. (G) Introduction of the reverse mutation into hSARM1^H583L^ results in strong increase in NADase activity and death in cultured HEK293F cells. Gray and black-striped bars show values gained 48 and 72 h post transfection, respectively. Three biological repeats, Student’s t test; ***p < 0.001; n.s.: no significance.

In the ceTIR-1 cryo-EM structure, eight TIR domains assemble into an active array in which each domain engages in contralateral and lateral interactions (Figures 5A and 5B), as previously revealed in the crystal structure and the recent cryo-EM structure of hSARM1^TIR^.^25,30^ The contralateral interactions are chiefly mediated by close homotypic contacts of the αA helices. The lateral interactions are made between the BB loop of one protomer and the βE strand of the next protomer, which stabilizes the BB loop in an open conformation that allows substrate binding. In this way, one NADase active site is formed by three neighboring protomers: the major protomer that harbors the catalytic glutamate residue (ceTIR-1 E788, homologs to hSARM1 E642), the lateral neighbor that holds the BB loop open, and the contralateral neighbor that projects a bE-aE loop into the active site (Figure 5B). We have examined three residues that are closely positioned next to the NAD^+^ binding site and are different between hSARM1 and ceTIR-1: ceTIR-1^F809^/hSARM1^W662^, ceTIR-1^R774^/hSARM1^K628^, and ceTIR-1^D834^/hSARM1^E686^. We hypothesized that one of these residues may have a dominant effect over the different enzymatic properties of ceTIR-1 and hSARM1 (in particular, *K*m). We therefore substituted these residues accordingly: ceTIR-1^F809W^, hSARM1^W662F^, ceTIR-1^R774K^, hSARM1^K628R^, and ceTIR-1^D834E^; and we expressed and purified them as we did with hSARM1 and ceTIR-1. We then measured their kinetics but found that none of these substitutions had any significant effect on *K*m (Figure S8).

As described above, one TIR domain is not assembled with the other eight TIRs (Figures 2F and 2G). We considered that this anomaly might be due to the odd number of protomers (9), where the ninth TIR domain can only form lateral contacts, without having a contralateral neighbor. Based on this observation, we hypothesized that in ceTIR-1, the affinity of TIR interaction with ARM is greater than the TIR-TIR lateral contact but weaker than the combined contralateral and lateral interactions. Unlike the constitutively assembled TIR array of ceTIR-1, the active TIR array in hSARM1 is transitory, and it assembles only in the presence of the NAD^+^ substrate or a non-hydrolysable inhibitor.^25^ We have noticed that a single residue substitution in the αA helix that mediates the contralateral interactions (ceTIR-1^L729^/hSARM1^H583^) expands the hydrophobic homotypic interface in ceTIR-1 and therefore is likely to strengthen this interaction (Figure 5C). To test this hypothesis, we first analyzed the oligomerization state of ceTIR-1^TIR^ in solution using size exclusion chromatography (Figure 5D) and crosslinking (Figure 5E) and found that ceTIR-1^TIR L729H^ is indeed considerably less dimerized than the non-mutated ceTIR-1^TIR^. Next, we found that in cultured cells, the reciprocal hSARM1^H583L^ mutation increases NADase activity and cell death, nearing to the levels of the strong activating mutation “delARM” (Figure 5F). Finally, for *in vitro* NADase measurements, we have compared time-dependent NAD^+^ consumption by CHIMERA to CHIMERA^L729H^ and found that, consistently, the L729H mutation attenuates NAD^+^ catalysis (Figure 5G).

Together, these experiments reveal how the ceTIR-1^L729^/hSARM1^H583^ side chains on the αA helix control auto-inhibition and NADase activity by forming stronger (in ceTIR-1) or weaker (in hSARM1) contralateral homotypic TIR-TIR interactions. Notably, the strong activating effect of the hSARM1^H583L^ mutation in cultured cells demonstrates the necessity of weaker TIR-TIR interactions in hSARM1 to avoid uncontrolled toxic NADase activity. In ceTIR-1, despite stronger contralateral homotypic TIR-TIR interactions, toxicity is diminished because of the weaker SAM domain oligomerization and weaker NADase activity.

### NAD^+^ consumption, but not cADPR production, determines toxicity in HEK293F cells

While NAD^+^ depletion^42^ is the primary effector of SARM function in WD, recent data suggest that in other contexts, the production of ADPR,^43^ cADPR,^44^ or other base-exchange products^11,18^ could mediate degeneration.

**Figure 6.**
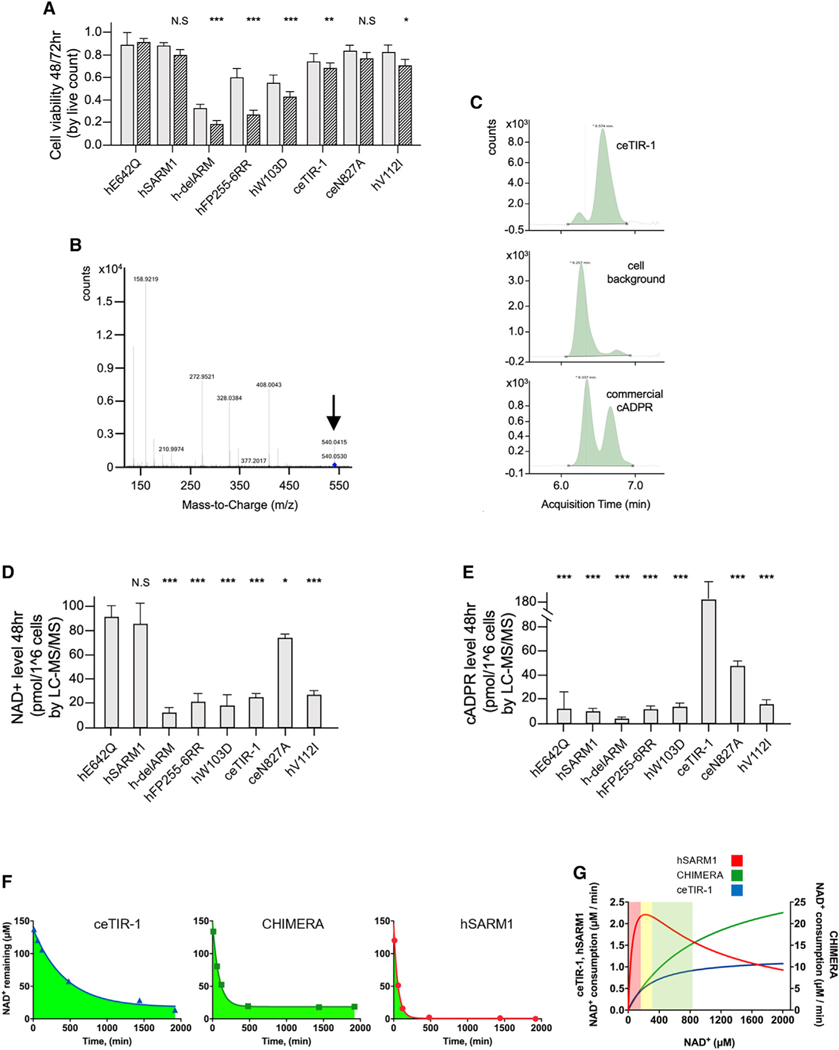
Extensive NAD^+^ consumption, but not cADPR production, leads to cell death in hSARM1/ceTIR-1 transfected cells (A) Cell death induced by hSARM1 and ceTIR-1 constructs show that hSARM1^V112I^ expression has a similar toxic effect over HEK293F cells as ceTIR-1 72 h post infection, but it has weaker effect than the strong activating mutations hSARM1^delARM^, hSARM1^FP255–6RR^, and hSARM1^W103D^. Gray and black-striped bars show values gained 48 and 72 h post transfection, respectively. (B) Mass spectrum fragmentation pattern by collision-induced dissociation of cADPR produced from expression of hSARM1/ceTIR-1 in HEK293F cells. Precursor ions are indicated by an arrow. (C) The commercial cADPR control (SIGMA #C7344) appears as a doublet acquisition-time peak. Also, the left, low-acquisition-time peak is dominant in non-transfected cells, while the right peak appears as a product of hSARM1 and ceTIR-1 activity. (D and E) Quantification of the LC-MS/MS measurements of NAD^+^ (D) and cADPR (E) levels in live cells. Three biological repeats, Student’s t test; ***p < 0.001; **p < 0.01; *p ˂ 0.05; n.s.: no significance. (D) There is 85% reduction in NAD^+^ levels by the most active hSARM1 construct, hSARM1^delARM^ and ~75% reduction by ceTIR-1 and hSARM1^V112I^. The mild loss-of-function mutation ceTIR-1^N827A^ has 25% reduced NAD^+^ levels. (E) Low cADPR basal levels appear in all hSARM1 transfected cells, including the gain-of-function mutants and the hSARM1^E642Q^ NADase-dead mutant. ceTIR-1 produces high levels of cADPR, 153 more than the basal level. Even the ceTIR-1^N827A^ loss-of-function mutant produces about 4× more. (F and G) *In vitro* NADase activity comparison between equal amounts (260 nM) of hSARM1, ceTIR-1, and CHIMERA, all supplemented with 75 μM NMN (F). 150 μM NAD^+^ was added and measured by HPLC over time. Data points represent the mean of three measurements. Note that while hSARM1 consumes virtually all the NAD^+^, both ceTIR-1 and CHIMERA leave 15–20 μM NAD^+^ intact, regardless of the initial difference in reaction rate between them. A likely explanation for that is the inherent difference in *K*m between the NADase activities of the hSARM1 and ceTIR-1 TIR domains, as represented in (G).

Thus, we explored the mechanism by which SARM can trigger cell death in HEK293 cells. To dissect this problem, we first searched for a mutation that will mildly activate hSARM1 to inflict similar levels of cell death in culture as ceTIR-1. This is because, unless activated, hSARM1 does not cause substantial cell death when recombinantly expressed in HEK293F cells due to inhibitory mechanisms that keep it from being active. Unlike hSARM1, we show here that ceTIR-1 does not seem to be subjected to cellular inhibition when recombinantly expressed in HEK293F cells and exhibits significant NAD^+^ consumption and cell death 48–72 h post infection (Figure 3E). Various hSARM1 mutations that alleviate inhibition lead to increased NAD^+^ consumption and early cell death. Weak activating mutations were recently discovered in ALS cohorts,^45,46^ most of which are located in the ARM domain. We found that while some hSARM1 mutations are too strong and others too weak, one mutation, V112I^26,45^ (although a significant activation by this mutation was not observed^46^), seems to inflict similar cell death level as ceTIR-1 in HEK293F (Figure 6A).

Next, to characterize the reaction products of these different versions of SARM, we used LC-MS/MS to assess the NAD^+^ and cADPR levels in cells transfected with hSARM1, hSARM1^E642Q^ (loss of function), hSARM1^delARM^ (very strong gain of function), hSARM1^W103D^ (strong gain of function), hSARM1^V112I^ (mild gain of function), and ceTIR-1 (Figures 6D and 6E). We found that 48 h post infection, hSARM1 does not show a significant NAD^+^ decrease when compared with the inactive hSARM1^E642Q^ control. ceTIR-1 and hSARM1^V112I^ bring about a 67% and 64% decrease in cellular NAD^+^ levels, respectively. ceTIR-1 produces far greater levels of cADPR than all hSARM1 constructs consistent with the *in vitro* measurements that are analyzed by high-performance liquid chromatography (HPLC) (Figures 3D and 3E).

These results lead to several conclusions. First, it provides further evidence for hSARM1 inhibition and for the uninhibited NADase activity of ceTIR-1 in HEK293F cells (although it may not be the case in *C*. *elegans* axons). Further, these data demonstrate that even a large decrease in NAD^+^ levels (by ceTIR-1 and hSARM1^V112I^) does not inflict immediate massive cell death. Only a near-complete NAD^+^ depletion, such as that executed by the stronger activating hSARM1 mutations, induces fast cell death. Finally, the comparison between ceTIR-1 and hSARM1^V112I^, where both inflict similar NAD^+^ loss and cell death, but the former produces about 90% cADPR and the latter 90% ADPR (and probably also related base-exchange products), implies that neither one of the products is more toxic than the other, leading to the conclusion that the extent of NAD^+^ depletion, and not the chemical composition of the products, determines cell toxicity.

Following this realization, we next monitored the *in vitro* consumption of 150 mM NAD^+^ over time by equal amounts (260 nM) of hSARM1, ceTIR-1, and CHIMERA, all supplemented with 75 mM NMN (Figure 6F). The reaction rate is fastest in hSARM1, which consumes all the detectable NAD^+^ by t = 500 min. The weakest activity is presented by ceTIR-1, which slows down continuously, leaving 60 μM NAD^+^ at t = 500 min and 15 μM by t = 2,000 min. The CHIMERA shows faster rate than ceTIR-1 at the first stage of the reaction, consistent with its higher *K*cat, but it quickly slows down to a halt at t = 500 min, leaving 20 μM NAD^+^ un-cleaved. This experiment exemplifies the impact of *K*m over the consumption of NAD^+^ at low concentrations (Figure 6G), where the low *K*m value of ~30 μM allows hSARM1 to consume almost all the NAD^+^, while the 10-fold higher *K*m values of ceTIR-1 and CHIMERA attenuate the reaction as it progresses, leaving 15–20 μM NAD^+^ intact, even after many hours.

## DISCUSSION

Wallerian axonal degeneration is strictly reliant on the NADase activity of SARM1. It is therefore intriguing that the endogenous ortholog TIR-1 does not support WD in *C*. *elegans*,^35^ despite its demonstrated NADase activity and overall high sequence similarity to SARM1. We find here that ectopic expression of hSARM1 is sufficient to promote *C*. *elegans* WD after axon injury, while equivalent expression of TIR-1 is not. We have performed structural and biochemical investigations and reported the differences between hSARM1 and ceTIR-1. We have observed several molecular differences in the following: (1) strength of oligomerization via SAM domains, (2) TIR NADase kinetic parameters, (3) dynamics of TIR oligomeric assembly, (4) strength of auto-inhibition, (5) regulation by nicotine nucleotides molecules, (6) chemical structures of TIR NADase products, and (7) TIR NADase effect over cell death.

We consider that the most important factor that explains the underlying functional discrepancy between hSARM1 and ceTIR-1 is *Km*. Once activated, hSARM1 has a NADase *K*m of about 30 mM^15,20,26,30^ (although Jiang et al.^14^ have calculated a 43 higher value), and here we have measured a *K*m of 250 mM for ceTIR-1 (although Loring et al.^31^ have calculated a lower value). Possibly, ceTIR-1 preserves the high *K*m of the ancestral bacterial TIR NADases.^47^ *K*m represents the relationship between the rate of reaction and concentration of substrate (*K*m is the substrate concentration that yields a half-maximal enzyme velocity), and as the NAD^+^ concentration drops further below *V*max due to continued consumption by the NADase activity, so decreases the rate of the reaction, according to the *K*m. For example, while at 500 mM NAD^+^ the turnover activity of ceTIR-1 is only 2-fold slower than hSARM1, it becomes 10-fold slower at 25 mM. Such a difference in *K*m would be physiologically relevant under energetic stress when the cellular NAD^+^ levels dwindle. In such conditions, hSARM1 will continue catalyzing NAD^+^ vigorously, while ceTIR-1 activity attenuates, without the ability to consume the NAD^+^ stores completely (Figure 6G).

Besides suggesting that hSARM1 and TIR-1 must have structural and biochemical differences that account for these differences in degeneration, our data suggest an overall functional divergence between *C*. *elegans* TIR-1 and its orthologs in humans and many other species. In *C*. *elegans*, the primary function of TIR-1 is apparently to support MAK kinase signaling,^29^ rather than to destroy NAD^+^ in response to injury signals. Exactly how ceTIR-1 performs this signaling role—and whether other SARM orthologs perform similar signaling functions in addition to their prominent role in degeneration—is an interesting question that may be informed by our structural studies. Our data indicate that expression of hSARM1 is sufficient to confer WD to *C*. *elegans*. However, unlike other systems, only some neurons expressing hSARM1 degenerate after injury, while others are preserved. Whether or not degeneration occurs does not appear to be a simple result of how much hSARM1 is expressed. Rather, unknown factors seem to determine the hSARM1-dependent outcome in each neuron. Identifying these factors may reveal additional aspects of the cell biology of WD.

## Conclusion

In conclusion, our investigation into the absence of WD in *C*. *elegans* has revealed that while hSARM1 ectopic expression in *C*. *elegans* neurons leads to axon degeneration following nerve injury, the ceTIR-1 does not. Together with structural and enzymatic studies *in vitro* and in cell culture, we have determined that the stronger NADase activity of hSARM1, particularly its lower *K*m value, is the fundamental reason behind its ability to induce axon degeneration, whereas ceTIR-1, with weaker activity, fails to do so. We propose that the disparities in cellular function and toxicity between these two orthologs dictate a series of alterations in structure, oligomerization, auto-inhibition, and product ratios that we have documented here. This study enhances our understanding of the molecular mechanisms and evolution of SARM orthologs and WD across different species.

Furthermore, we are introducing a new *C*. *elegans* transgene tool, named XE3145, which holds promising potential for future research in axon degeneration and regeneration studies within the *C*. *elegans* model.

### Limitations of the study

This study does not specifically address the physiological function of ceTIR-1. For example, although we have demonstrated that ceTIR-1 is not significantly inhibited by NAD and does not activate in response to NMN, we have yet to elucidate the underlying mechanisms that govern ceTIR-1 NADase activity. Additionally, there are two primary limitations that warrant consideration. Firstly, it is essential to assess the extent to which the *in vitro* results accurately reflect the *in vivo* behavior of ceTIR-1. While we have demonstrated that ceTIR-1 ring assembly is comparatively weaker than that of hSARM1, it remains unclear whether this disparity represents an inherent property or merely an experimental artifact resulting from the *in vitro* conditions. To address this concern, we have developed the CHIMERA construct and verified that even when assembled within the stable scaffold of the hSARM1 SAM ring, the ceTIR-1 TIR domains exhibit weak NADase activity and low cellular toxicity, as evidenced by the CHIMERA^FP255–6RR^ gain-of-function mutant. Secondly, our findings indicate that hSARM1 induces degeneration in 37% of *C*. *elegans* axons, while its effects in mouse models are more pronounced. Notably, it does not appear that degeneration is solely determined by the level of hSARM1 expression. Instead, unidentified factors appear to dictate the outcome of hSARM1-dependent degeneration in each neuron. Unraveling these factors could provide valuable insights into additional aspects of the cellular biology underlying WD.

## STAR★METHODS

### RESOURCE AVAILABILITY

#### Lead contact

Further information and requests for resources and reagents should be directed to and will be fulfilled by the lead contact, Yarden Opatowsky yarden.opatowsky@biu.ac.il.

#### Materials availability

All unique/stable reagents generated in this study are available from the lead contact with a completed materials transfer agreement.

#### Data and code availability

- Coordinates and map have been deposited in the Protein DataBank and in the EMDB with the following accession codes: PDB 8P2L and EMD-17369 for CHIMERA, PDB 8P2M and EMD-17370 for ceTIR-1.
- This paper does not report original code.
- Any additional information required to reanalyze the data reported in this work is available from the lead contact upon request.

### EXPERIMENTAL MODEL AND STUDY PARTICIPANT DETAILS

#### Animals

##### Plasmids

Sequences encoding human and *C*. *elegans* SARM orthologs were cloned into Gateway [1–2] entry vectors via Gibson Assembly. To build [1–2] hSARM1, codon-optimized hSARM1 with artificial introns was synthesized as a gBlock (IDT), and the [1–2] entry vector sequence was amplified with YW462/463 before Gibson assembly. To build [1–2] ceTIR-1, a generic intron was inserted into *tir-1* cDNA in the [1–2] Gateway entry vector after nucleotide 86 via Q5 mutagenesis protocol using primer set CF15/16, after Gibson assembly. Final plasmids were generated by multisite Gateway LR (Invitrogen), using the [1–2] components described above, together with [4–1] *Pitr-1* promotor, [2–3] SL2mCherryH2Blet858 3′UTR, and pDest r4-r3 vector.

#### Worm strains

Animals were maintained under standard conditions and were raised at 20°C on NGM plates seeded with OP50 *E*. *coli*. The strains listed in Table S2 were made by microinjection into N2 worms. Primers used in vectors construction are listed in Table S3.

#### Laser axotomy

Laser induced axon severing was performed as described preciously.^39^ Briefly, L4 animals were immobilized with 0.05 μM polystyrene beads on a 3% agarose pad between a glass slide and a coverslip. Using a Nikon Eclipse 80i microscope equipped with a 100x NA Plan Apo VC lens, DA9 axons were severed by 10 pulses from a 435 nM Micropoint laser at 20 Hz, targeting the axon at the dorsal-ventral midline.

#### Fluorescence microscopy

To evaluate axon degeneration, worms were imaged 18h after axon injury. Worms were immobilized with 8mM levamisole and mounted on a 3% agarose pad between a slide and coverslip. Z stacks with 0.2 μM steps were taken using MetaMorph software (Molecular Devices) under a Leica DMi8 microscope equipped with a Visitech i-SIM super-resolution confocal system, an ASI-XYpZ Piezo stage and a Hamamatsu ORCA-Flash4.0 CMOS camera. 300ms exposure time were used for both 488nm channel and 561nm channel. Maximum projections were generated with ImageJ. Assessment of degeneration was made based on maximum projections as well as original z stacks. Fluorescence intensity (FI) of mCherryH2B was measured based on maximum projections in the DA9 nucleus, normalized by subtracting background FI, and plotted in Prism 7.0 software.

### METHOD DETAILS

#### cDNA generation and subcloning

Cloning of all the constructs was made as detailed in^15^ by PCR amplification from the complete cDNA clone (Imagene) of ceTIR-1 (uniport: Q86DA5) and hSARM1 (uniport: Q6SZW1).

For bacterial expression, ceTIR-1^TIR^ (^703^MLS . QQR^872^) was ligated into pET28 modified to contain N-terminal His tag followed by a TEV digestion sequence.

For expressions in mammalian cell culture, several constructs of hSARM1 and ceTIR-1 were generated: the near-intact hSARM1 (^26^ERL . GPT^724^) and the mutants hSARM1^delARM^, hSARM1^FP255–6RR^, hSARM1^W103D^, hSARM1^E642Q^, hSARM1^W662F^, hSARM1^K628R^, hSARM1^H853L^, and hSARM1^V112I^, the near-intact ceTIR-1 (^162^HMD . QQR^872^) and the mutants ceTIR-1^FF407–8RR^, ceTIR-1^E788Q^, ceTIR-1^F809W^, ceTIR-1^R774K^, ceTIR-1^D834E^ and ceTIR-1^L729H^.

In addition, we have generated by assembly PCR a CHIMERA construct with human ARM and SAM domains and *C*. *elegans* TIR (h^26^ERL . DTP^562^-ce^709^DVF … QQR^872^).

All the constructs were ligated into a modified pEGFP-N1 mammalian expression plasmid that is missing the C terminus GFP fusion protein and includes N-terminal 6*HIS-Tag followed by a TEV digestion sequence. Assembly PCR mutagenesis (based on https://openwetware.org/wii/Assembly_pcr) was used to introduce all the point mutations.

#### Protein expression and purification

For bacterial expression and purification, ceTIR-1^TIR^ was expressed in the T7 express E. coli strain (NEB), also containing the RIL codon plus plasmid. The construct were transformed into E. coli and grown for 2–4 h at 37°C. The media used for ceTIR-1^TIR^ was 2YT containing 34μg/mL chloramphenicol and 50 μg/mL Kanamycin. When the culture reached an OD600 = 0.6–0.8 protein expression was induced with 200 mM IPTG over a 14–16 h period at 16°C. After overnight growth, the cells were harvested and frozen in liquid nitrogen prior to lysis. Cells were suspended at a 1:8 (w:v) ratio with lysis buffer (50mM Phosphate buffer (pH 8), 400mM NaCl, 5% glycerol, 1mM EDTA, 5mM β-mercaptoethanol) and lysed using a microfluidizer (Microfluidics). 2mM PMSF were added immediately after lysis and cell debris were removed twice by 20 min of centrifugation (10,000g) at 4°C. The supernatant was then loaded on pre-equilibrated nickel-chelate column (HisTrap) with buffer A (50mM Phosphate buffer (pH 8), 400mM NaCl, 5% glycerol, 5mM β-mercaptoethanol) at a flow rate of 2.5 mL/min; next the column was washed with 4% of buffer B (50mM Phosphate buffer (pH 8), 400mM NaCl, 5% glycerol, 5mM, 0.5mM imidazol β-mercaptoethanol) until stable baseline was achieved, and protein was eluted by applying 40–250 mM imidazole buffer B gradient. Protein containing fractions were pooled and incubated at 4°C with 1:50 w/w TEV protease for overnight dialysis against buffer A. The next day, protein containing fractions were centrifuged at 10,000 g for 10 min and loaded again on the pre-equilibrated nickel-chelate column with buffer A. Then, the column was washed and eluted with a gradient of buffer B. The relevant fractions were pooled and concentrated using a spin concentrator and then loaded onto pre-equilibrated superdex HiLoad 26/600 (GE Healthcare) for size exclusion chromatography. The elution was performed with 20mM HEPES (pH 7.4), 1% glycerol, 300mM NaCl and 1mM DTT. Protein-containing fractions were pooled and concentrated using a spin concentrator. The concentrated proteins were split into aliquots, flash-frozen in liquid N_2_ and stored at −80°C for later enzymatic assays.

For mammalian expression and purification, hSARM1, hSARM1^W662F^, hSARM1^K628R^, ceTIR-1, ceTIR-1^E788Q^, ceTIR-1^F809W^, ceTIR-1^R774K^, ceTIR-1^D834E^, ceTIR-1^L729H^, and CHIMERA were expressed in HEK293F suspension cell culture, grown in FreeStyle 293 medium (GIBCO), at 37°C and in 8% CO_2_. Transfection was carried out using preheated (70°C) 40 kDa polyethyleneimine (PEI-MAX) (Polysciences) at 1mg of plasmid DNA per 1 L of culture once cell density has reached 1*10^6^ cells/ml. Expression levels were assessed by anti-FLAG WB 18 h post transfection, showing similar levels of expression in all constructs that are not strong activators of hSARM1. Cells were harvested 4 days after transfection by centrifugation (10 min, 1500 × g, 4°C), re-suspended with buffer A (50mM Phosphate buffer pH 8, 300mM or 120mM NaCl (for human or C. elegans construct respectively),5% glycerol, 1mM DTT, 0.5mM EDTA, protease inhibitor cocktail from Roche) and lysed using a microfluidizer followed by two cycles of centrifugation (12000 × g 20 min). Supernatant was then filtered with 45 mM filter and loaded onto a pre-equilibrated Ni-chelate column. The column was washed with buffer A supplemented with 25mM Imidazole until a stable baseline was achieved. Elution was then carried out in one step of 175mM Imidazole, after which protein-containing fractions were pooled and loaded onto pre-equilibrated Superdex 200 Increase 10/300 (GE Healthcare) for size exclusion chromatography and elution was performed with 25mM Phosphate buffer pH 8.5, 120 mM NaCl, 2.5% glycerol, and 1mM DTT. Protein-containing fractions were split into aliquots, flash-frozen in liquid N_2_ and stored at −80°C for later cryo-EM visualization and enzymatic assays.

#### HPLC analysis of NADase activity

For quantification of hSARM1, ceTIR-1, ceTIR-1^TIR^, hSARM1^W662F^, hSARM1^K628R^, ceTIR-1^F809W^, ceTIR-1^R774K^ ceTIR-1^D834E^ and CHIMERA NADase activity, purified proteins were first diluted to 533 nM concentration in 25 mM HEPES pH 7.5, 150mM NaCl and 1:100 (v/v) BSA (NEB Inc, 20 mg/mL), and then mixed with different concentrations of NAD^+^ (in the same buffer) in a 1:1 v/v ratio. All the ceTIR-1 constructs were incubated at 27°C for 30 min. All the hSARM1 and CHIMERA constructs were incubated at 37°C for 10 min. The reactions were stopped by adding an equal volume of 0.4% formic acid and centrifuged at 12000g for 2 min at room temperature.

HPLC measurements were performed using a Merck Hitachi Elite LaChrom HPLC system equipped with an autosampler, UV detector, and quaternary pump. HPLC traces were monitored at 260 nM and integrated using EZChrom Elite software. Ten microliters of each sample were injected onto a Waters Spherisorb ODS1 C18 RP HPLC Column (5 μm particle size, 4.6 mm × 150 mm). HPLC solvents are as follows: A: 100% methanol; B: 120mM sodium phosphate pH 6.0; C double-distilled water (DDW). The column was pre-equilibrated with B:C mixture ratio of 80:20. Chromatography was performed at room temperature with a flow rate of mL/min. Each analysis cycle was 12 min long as follows (A:B:C, v/v): fixed 0:80:20 from 0 to 4 min; gradient to 20:80:0 from 4 to −6 min; fixed 20:80:0 from 6 to 9 min, gradient to 0:80:20 from 9 to 10 min; fixed 0:80:20 from 10 to 12 min. The NAD^+^ catalysis products ADPR and cADPR are eluted at the isocratic stage of the chromatography while NAD^+^ and NAM elute in the methanol gradient stage.

#### Resazurin assay

HEK293F cells were seeded in 24-well plates (1 million cells in each well) in a final volume of 1 mL of FreeStyleTM 293 medium (GIBCO). The cells were transfected with preheated (70°C) linear polyethlenimine (PEI) at 1 μg DNA of different hSARM1 and ceTIR-1 constructs and incubated in an orbital shaker at 37°C, 120 rpm and 8% CO_2_. After 24hr, 100μL of cells were collected from each well every day for 3 days and centrifuged at 600g for 10 min at room temperature. The pelleted cells were then re-suspended with 0.03 mg/ml Resazurin sodium salt (SIGMA) dissolved in FreeStyleTM 293 medium (GIBCO). All the samples were incubated for 45 min at 37°C, centrifuged (10min, 600g, 25°C) and then transferred to a 384-well black plate (Corning). Fluorescent data were measured using a SynergyHI (BioTek) plate reader at 560 nM excitation and 590 nM emission wavelengths. All fluorescent emission readings were averaged and normalized by subtracting the Resazurin background (measured in wells without cells). Three repeats were performed for each construct with mean ± SEM.

#### Cell viability assay

HEK293F suspension cells culture were seeded in 24-well plates (1 million cells in each well) in a final volume of 1 mL of FreeStyleTM 293 medium (GIBCO). The cells were transfected with preheated (70°C) linear polyethlenimine (PEI) at 1 mg DNA as described before and incubated in an orbital shaker at 37°C, 120 rpm and 8% CO_2_. Live cells were counted using the trypan blue viability assay and an automatic cell counter (NanoEnTek) every 24 h for 3 days. Data was presented as mean ± SEM for two repeats of each construct.

#### Sample preparation for LC-MS/MS

HEK293F cells were seeded in 24-well plates (1 million cells in each well) in a final volume of 1 mL of FreeStyleTM 293 medium (GIBCO). The cells were transfected with 1 μg DNA as described before and incubated at 37°C and in 8% CO_2_. When required, the cells were collected and pelleted by centrifuged at 1000g for 10 min at 4°C and aliquots were saved for cell counting using the trypan blue viability assay. Pelleted cells were washed with 1mL PBS X1, centrifuged at 1000g for 10 min at 4°C and then stored in liquid nitrogen prior to metabolite extraction. Extraction of the desired metabolites from cells were performed as in.^58^ 1X10^6 HEK293-F cell pellets were dissolved in 140μL of preheated (80°C) buffered ethanol solution (3:1) (ethanol:HEPES [1mM, pH 7.4]). For control, a standard metabolite mix was used. Samples were then centrifuged at 13,200g for 15 min at 4°C and then the supernatants were collected for LC-MS/MS analysis.

#### LC-MS/MS metabolites measurement

All the equipment for the analyses was from Agilent technologies and consisted of a 6545 QTOF mass spectrometer equipped with an electrospray ionization interface (ESI) coupled to a 1260 UHPLC, a G4204A quaternary pump, G4226A ALS auto-sampler, and G1316C thermostatted column compartment. UHPLC was carried out on a SeQuant ZIC-cHILIC 3 μM, 100Å 100 × 2.1 mm column with water (0.1% formic acid)-MeCN gradient elution, from 5 to 95% MeCN for 10 min at a flow rate of 0.5 mL/min. Twenty μL of each sample and standards were injected into the LC-MS/MS instrument in triplicate and an average peak area of three analyses was calculated. A water: MeCN (1:1) solution was injected as a blank within a sequence of samples to confirm that there was no cumulative carryover. Mass spectral parameters were optimized for each compound by varying the fragmentor voltage of the ion source for scan mode and collision energy for product ion mode (MS/MS). Specific parameters of the ion source were readjusted. The ESI was operated in positive mode for NAD^+^ and NAM and in negative mode for ADPR and cADPR. Detection of NAD^+^ and NAM were monitored by the ions transitions 664.115 m/z [M + H] ^+^→ 428.036 m/z and 123.055 m/z [M + H]^+^ → 53.038 m/z (respectively), energy for product ion mode (MS/MS) 25 and 45 V. ADPR and cADPR were monitored by the ions transitions 558.064 m/z [M-H]^−^→ 346.057 m/z and 540.053 m/z [M-H]^−^→ 158.952 m/z (respectively). Positive and Negative ESI was operated as follow: fragmentor 250 V, source temperature was set to 300°C, drying gas 8 L/min, nebulizer 40 psi, sheath gas temperate 400°C, sheath gas flow 12 L/min 400°C, ion spray voltage was 3.5 kV and collision, energy for product ion mode (MS/MS) 35 V for both.

#### Quantification of NADase metabolites

To determine the concentration of the investigated metabolites, calibration curves were generated with synthetic cADPR (SIGMA C7344), ADPR (SIGMA A0752), NAD^+^ (SIGMA NAD100-RO) and NAM (SIGMA 72340). Quantification of these metabolites in the HEK293F cells was calculated from the standard curve and presented as a mole of molecules per million cells.

#### Calculation of kinetic parameters’ values

For *V*_max_, *K*_m_ and *K*_i_ determination, the NADase activity assay was performed with several different NAD^+^ substrate concentrations and sampled in constant time points. For each NAD^+^ concentration, linear increase zone was taken for slope (*V*_0_) calculation. All data were then fitted to the Michaelis-Menten equation or to substrate inhibition equation using non-linear curve fit in GraphPad software. *K*_cat_ was calculated by dividing the *V*_max_ with protein molar concentration.

#### Determination of *IC*_*50*_

Serial concentrations of nicotinamide (NAM), a known inhibitor of hSARM1 were pre-incubated with 260 nM of ceTIR-1 or with 37.39μM ceTIR-1^TIR^ for 10 min at room temperature, followed by the addition of 150uM NAD or 300 μM NAD, respectively. After incubation of 60 min for ceTIR-1 and of 10 min for ceTIR-1^TIR^ at 27°C, the reactions were stopped by adding an equal volume of 0.4% formic acid and centrifuged at 12000g for 2 min at room temperature. The amount of cADPR, ADPR products and NAD consumption were measured by HPLC and used to calculate the *IC*_*50*_ values by plotting the normalize response to the dose of the NAM (log10). All calculations were performed in GraphPad Prism software.

#### Temperature-preference NADase assays

To determine the optimal temperature for NADase activity, hSARM1 and ceTIR-1 were diluted to a final concentration of 553 nM. The enzymes were incubated with 150μM NAD (final concentration) at various temperatures (7°C, 12°C, 17°C, 22°C, 27°C, 32°C, 37°C, 42°C) for 30 min. All the reactions were stopped by adding an equal volume of 0.4% formic acid, centrifuged at 12000g for 2 min at room temperature and analyzed by HPLC.

#### Time-dependent NADase activity assays

To measure the NADase activity of hSARM1, ceTIR-1 and CHIMERA over time, equal amount of the enzymes (260 nM) were pre-incubated with 75μM NMN (SIGMA N3501) for 10 min. The reactions were initiated by addition of 150μM NAD and incubated for different time lengths - 10, 60, 120, 480,1140 and 1920 min. All the reactions were stopped by adding an equal volume of 0.4% formic acid and centrifuged at 12000g for 2 min at room temperature. NAD consumption was determined by HPLC.

#### ceTIR-1^TIR^ cADPR/ADPR ratio assays

To determine the ratio between cADPR to ADPR production, we diluted ceTIR-1^TIR^ to 6 different concentrations from 74.78 μM to 1.8696 μM and then mixed with a constant final concentration of 500 μM NAD. The reactions were incubated for 5, 30 and 60 min. All the reactions were stopped by adding an equal volume of 0.4% formic acid and centrifuged at 12000g for 2 min at room temperature. The reactions were measured based on the rate of NAD consumption and by cADPR and ADPR production with HPLC.

#### NADase activity of SARM1 proteins in the presence of NMN

Equal concentrations (260 nM) of hSARM1, ceTIR-1, ceTIR-1^TIR^ and CHIMERA were pre-incubated with different concentrations of NMN (0–250 μM) for 10 min and then 150 μM of NAD was added to each reaction. The reactions were stopped after 30 min by adding an equal volume of 0.4% formic acid and centrifuged at 12000g for 2 min at room temperature. NAD consumption was determined by HPLC.

#### HPLC chromatograms

The products were first identified separately by injecting each standard molecule. The retention time for ADPR is at ~ 4.5 min and for cADPR is at ~ 2.7 min.

The HPLC NADase profiles of hSARM1 and ceTIR-1 were generated by incubating equal amounts (260 nM) of each protein with 150uM NAD for 120min. The ceTIR-1^TIR^ profile was made by incubating 74.78μM of protein with 500uM NAD for 30 min.

#### Mass photometry

Mass photometry data was acquired using a One^MP^ mass photometer (Refeyn Ltd, Oxford, UK). The procedure used was based on a previous published protocol.^59^ Briefly, washed, and dried gaskets (with chambers for holding the protein solution) was assembled on a clean coverslip. 16 μL of 50 mM HEPES buffer was loaded in one of the chambers and used for focus. Next, 4 μL of protein solution (calibrant/test protein) was added and movie was recorded for a period of 120 s. A 50 nM solution of urease was used for mass calibration. Prior to mass photometry measurements, hSARM1 and ceTIR-1 were diluted with HEPES buffer to the final concentration of 50 nM. The movies were processed and analyzed using Discover^MP^.

#### Cryo-EM grids preparation

Cryo-EM grids were prepared by applying 3 μL protein samples to glow-discharged (PELCO easiGlow Ted Pella Inc., at 15 mA for 1 min) holey carbon grids (Quantifoil R 1.2/1.3, Micro Tools GmbH, Germany). The grids were blotted for 4 s and vitrified by rapidly plunging into liquid ethane at −182°C using Leica EM GP plunger (Leica Microsystems, Vienna, Austria). The frozen grids were stored in liquid nitrogen until the day of cryo-EM data collection.

#### Cryo-EM data acquisition and processing

For optimization of sample preparation, we used F30 Polara microscope in Ben-Gurion University, Israel. Samples were imaged under low-dose conditions on a FEI Tecnai F30 Polara microscope (FEI, Eindhoven) operating at 300kV. Datasets were collected using SerialEM^53^ on a K2 Summit direct electron detector fitted behind an energy filter (Gatan Quantum GIF) with a calibrated pixel size of Å. The energy filter was set to remove electrons > ±10eV from the zero-loss peak energy. The defocus range was set from −1.0μm to −2.5μm. The K2 summit camera was operated in counting mode at a dose rate of 8 electrons/pixel/second on the camera. Each movie was dose fractionated into 50 image frames, with total electron dose of 80e^−^/Å^2^. Dose-fractionated image stacks were aligned using MotionCor2,^54^ and their defocus values estimated by Gctf.^60^

For grid screening prior to large data collection, we used the Talos Glacios microscope in EMBL, Grenoble, France. Frozen grids were loaded onto a 200kV Talos Glacios (ThermoFisher) electron microscope equipped with a Falcon3 direct electron-counting camera (ThermoFisher). Cryo-EM data were acquired with EPU software (FEI) at a nominal magnification of 120,000, with a pixel size of 1.224Å, in linear mode and a total dose of ~44 electrons per Å2. Usually, 500–1000 micrographs were collected from each sample for initial evaluation. Preprocessing was performed in Warp pipeline including motion correction, CTF estimation and particle picking.^55^ Further data processing was conducted using the cryoSPARC suite, including 2D classification, ab-initio reconstruction and refinement.

For data collection of ceTIR-1 and CHIMERA, we used the Titan Krios microscope in ESRF CM01 beamline^56^ at Grenoble, France. Frozen grids were loaded into the microscope equipped with a Gatan K3 direct electron detector and a Gatan Bioquantum LS/967 energy filter. Cryo-EM data were acquired with EPU software (FEI) at a nominal magnification of ×105,000, with a pixel size of 0.839 Å. 6512 movies of ceTIR-1 and 7558 movies of CHIMERA grid sample were acquired in superresolution mode at a flux of 13.3 electrons per p^2^ s^−1^, giving a total exposure of 41.4 electrons per Å^2^ and fractioned into 40 frames. A defocus range from −0.8μm to −2.8 μm were used. Using the SCIPION wrapper^57^ the imported movies were drift-corrected using MotionCor2 and CTF parameters were estimated using Gctf for real-time evaluation. Further data processing was conducted using the cryoSPARC suite. Movies were motion-corrected and contrast transfer functions were fitted. Templates for auto-picking were generated by 2D classification of auto picked particles. For the ceTIR-1 data, after initial selection, template-based auto-picking produced a total of 370,021 particles, from which 121,487 were selected based on iterative reference-free 2D classifications for reconstruction of the ceTIR-1 structure. In the case of the CHIMERA data, a total of 298,564 particles were picked, from which 136,346 were selected based on iterative reference-free 2D classifications for reconstruction. Initial maps of both ceTIR-1 and CHIMERA were calculated using Ab-initio reconstruction. In the case of CHIMERA, high-resolution map was obtained by imposing C8-symmetry in non-uniform 3D refinement, while no symmetry was imposed in the case of ceTIR-1. Working maps were locally filtered based on local resolution estimates.

#### Model building and refinement

N-terminal domains of our previously determined hSARM1 NAD octamer structure (PDB 7ANW) and the alphafold model^40^ of ceTIR-1 TIR domain were positioned into the duplex CHIMERA map. Regions which had no clear density were truncated. Initial refinement was performed using PHENIX^48^ RealSpaceRefine^49^ program. The model was rebuilt in Coot^51^ and Isolde^61^ implemented in ChimeraX.^52^ The model was refined in Refmac5.^50^

The alphafold model of complete ceTIR-1 was docked into the C1 map. After fitting of ARM and SAM domains the predicted position of the TIR domain matched the map for only one of nine monomers. Remaining eight TIR domains formed a C2 symmetry complex on top of the C9 nanomer ring formed by ARM and SAM domains of ceTIR-1. The TIR domain octamer model was further predicted by Alphafold and positioned into the map. The model was rebuilt using Coot and Isolde and refined in Refmac5.

### QUANTIFICATION AND STATISTICAL ANALYSIS

The statistical significance: Student t test, Kolmogorov-Smirnov test, and Fisher’s exact test were used as indicated in the paper.

## Supplementary Material

1

2

3

4

## Figures and Tables

**Table:** KEY RESOURCES TABLE

REAGENT or RESOURCE	SOURCE	IDENTIFIER
Bacterial and virus strains		

T7 Express E. coli	NEB	C2566I

Chemicals, peptides, and recombinant proteins		

Tev protease with N terminal 6xHIS	This paper	N/A
ceTIR1 ^TIR^ (residues 703–872)	This paper	N/A
hSARM1 (residues 26–724) WT, various mutants.	This paper	N/A
With N terminal 6xHIS + TEV digestion site		
ceTIR1 (residues 162–872) WT, various mutants.	This paper	N/A
With N terminal 6xHIS + TEV digestion site		
hSARM1^delARM^ (residues 387–724) with N	This paper	N/A
terminal 6xHIS + TEV digestion site		
Chimera construct (residues hSARM1	This paper	N/A
26–562 -ceTIR1 709–872) with N terminal		
6xHIS + TEV digestion site		
cOmplete(TM), Mini, EDTA-free Protease I	Roche	11836170001
NAD^+^	SIGMA	NAD100-RO
ADPR	SIGMA	A0752
cADPR	SIGMA	C7344
NAM	SIGMA	72340
NMN	SIGMA	N3501
Kanamycin sulfate	Carl Roth Gmbh	25389–94-0
IPTG	ORNAT	INA-1758–1400
FreeStyleTM 293 expression medium	Thermo Fisher	12338018
polyethylenimine (PEI)	Polysciences,Inc.	24765–1
chloramphenicol	Carl Roth Gmbh	Cat# 56–75-7
Resazurin sodium salt	SIGMA	R7017
HEPES (2-[4-(2-Hydroxyethyl)	ApolloScientific	BI8181
piperazin-1-yl]ethanesulpho)		

Deposited data		

Chimera construct (residues hSARM1	This paper	PDB: 8P2L
26–562 -ceTIR1 709–872)		EMD-17369
ceTIR1	This paper	PDB: 8P2M
		EMD-17370

Experimental models: Cell lines		

HEK293F	Thermo Fisher Scientific	Cat#R79007

Recombinant DNA		

Modified pET28 (N-terminal His tag + TEV	This paper	N/A
digestion sequence)		
Modified pEGFP-N1 (N terminal 6xHIS + TEV	Sporny, M. et al. 2020^15^	N/A
digestion sequence and missing the C		
terminus GFP fusion protein)		
ceTIR1 ^TIR^ (residues 703–872) in	This paper	N/A
modified pET28		
hSARM1 (residues 26–724) WT and various	This paper	N/A
mutants in modified pEGFP-N1		
ceTIR1 (residues 162–872) WT and various mutants in modified pEGFP-N1	This paper	N/A
hSARM1^delARM^ (residues 387–724) in modified pEGFP-N1	This paper	N/A
Chimera construct (residues hSARM1 26–562 -ceTIR1 709–872) in modified pEGFP-N1	This paper	N/A

Software and algorithms		

PHENIX	Liebschner, D. et al.,2019^48^	N/A
RealSpaceRefine	Afonine, P. V. etal.,2018^49^	N/A
REFMAC5	Murshudov, G. N. et al.,2011^50^	N/A
Coot	Emsley, P. et al., 2010^51^	N/A
ChimeraX	Pettersen, E. F. et al., 2021^52^	N/A
Isolde	Croll, T. 2018	N/A
HPLC system	shimadzu corp.	Prominence-I LC-2030C plus
SynergyHI	BioTek	N/A
SerialEM	Mastronarde, D. N., 2005^53^	N/A
MotionCorr2	Li, X. etal., 2013^54^	N/A
Gctf	Zhang, K., 2016^55^	N/A
automatic cell counter	NanoEnTek	N/A
Mass Spectrometer	Agilent technologies	6545 QTOF
One^MP^ mass photometer	Refeyn Ltd, Oxford, UK	N/A
Discover^MP^		Version 2.3
glow-discharger	PELCO easiGlow™ Ted Pella Inc	N/A
Leica EM GP plunger	Leica Microsystems, Vienna, Austria	N/A
FEI Tecnai F30 Polara microscope	Eindhoven	N/A
Electron microscope	ThermoFisher	200kV Talos Glacios
electron-counting camera	ThermoFisher	N/A
EPU software	FEI	N/A
Titan Krios microscope	Kandiah, E. etal. 2019^56^	ESRF CM01 beamline
K3 direct electron detector	Gatan	N/A
SCIPION wrapper	Martinez, M. et al., 2020^57^	N/A
GraphPad Prism		Version 9

Other		

ceTIR1 cDNA	Bio basic	SYZ875–1
hSARM1cDNA	ImaGenes	uniport: Q6SZW1
pEGFP-N1 plasmid	Clontech	N/A
Spherisorb ODS1 C18 RP HPLC Column	Waters	PSS830613
HiLoad 26/600 Superdex 200 pg	GE Healthcare	Cat# 28989336
HiLoad 10/300 Superdex 200 increase	GE Healthcare	Cat# 28990944
HisTrap HP	GE Healthcare	Cat# 17524801
Holey carbon grids	Micro Tools GmbH, Germany	Quantifoil R 1.2/1.3,
Energy filter	Gatan	Quantum GIF
Energy filter	Gatan	Bioquantum LS/967

## Highlights

- Cryo-EM structure of the intact *C*. *elegans* SARM ortholog TIR-1 at 3.8 Å
- Expression of ceTIR-1 in *C*. *elegans* cannot induce axonal degeneration after injury
- Expression of human SARM1 in *C*. *elegans* induces axonal degeneration after injury
- Weaker NADase activity of ceTIR-1 might be the reason for functional disparity
