# Supplementary material for: Structure-function analysis of ceTIR-1/hSARM1 explains the lack of Wallerian axonal degeneration in C. elegans
Source: Cell Rep. Author manuscript; Available in PMC 2023 Nov 25. (PMC10675840; doi:10.1016/j.celrep.2023.113026)
Supplement: 1 [file NIHMS1933881-supplement-1.pdf]

Cell Reports, Volume *42*

Supplemental information

Structure-function analysis of ceTIR-1/hSARM1 explains the lack of Wallerian axonal degeneration in *C. elegans*

Tami Khazma, Atira Grossman, Julia Guez-Haddad, Chengye Feng, Hadas Dabas, Radhika Sain, Michal Weitman, Ran Zalk, Michail N. Isupov, Marc Hammarlund, Michael Hons, and Yarden Opatowsky

Figure S1

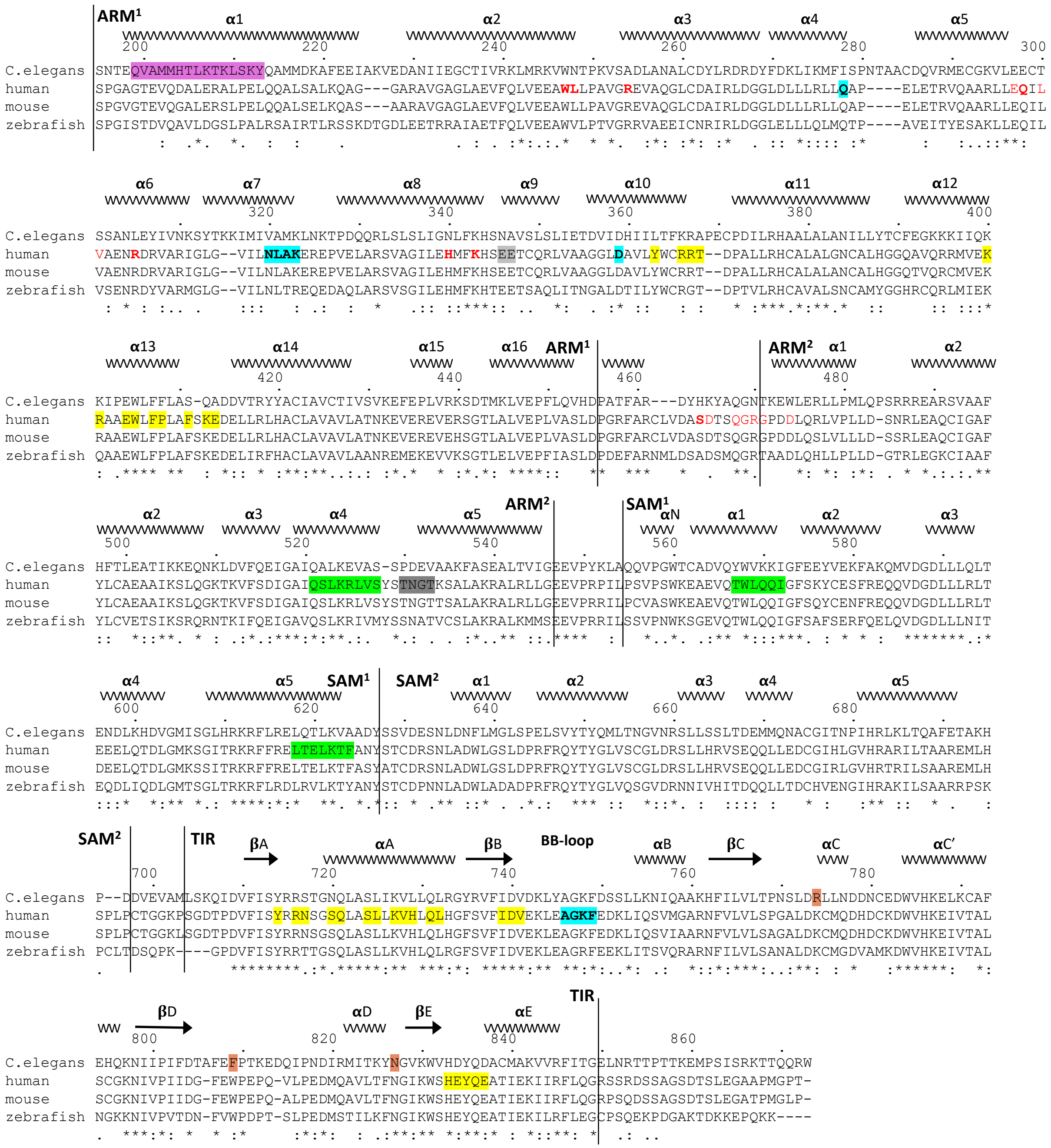

human|Q6SZW1
mouse|Q6PDS3
zebrafish|F1QWA8
C.elegans|Q86DA5-2

ARM-SAM interaction site
primary ARM-TIR docking site
secondary ARM-TIR docking site - **bold**
ARM-ARM interaction site - underscore
ARM-SAM same-chain interaction site
ARM NAD binding residues

Figure S1. Structure-based sequence alignment of the SARM1 of human, mouse, zebrafish, and the *C. elegans* homolog TIR-1. Color-coded highlights and Uniprot protein accession numbers are listed below.

Figure S2

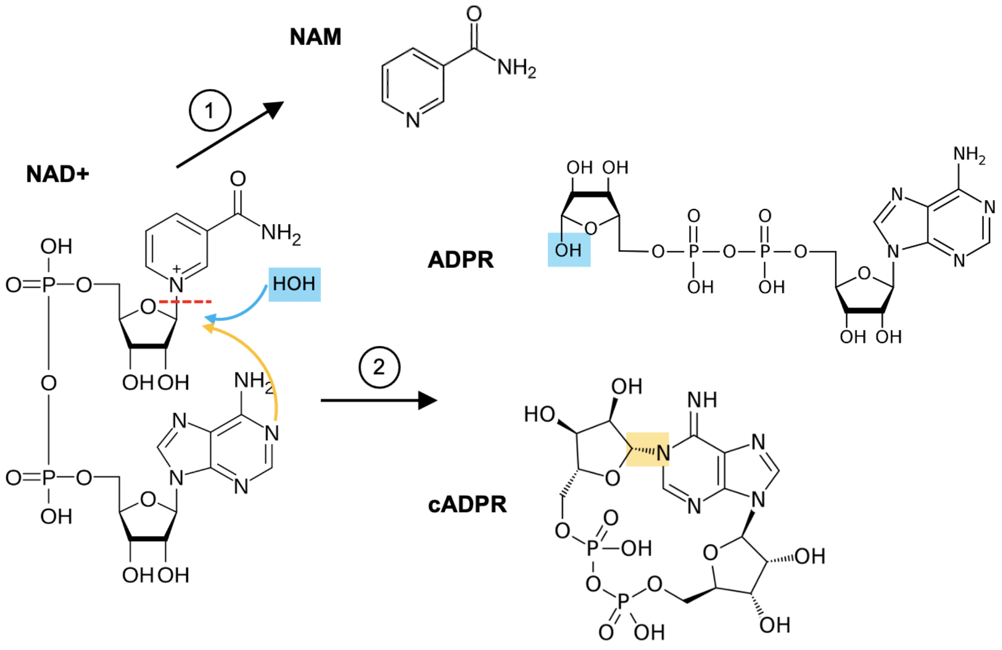

Figure S2. Simplified representation of NAD+ catalysis by SARM1/TIR-1.

Following a nucleophile attack by hSARM1E642/ceTIR-1E788 on the NAD+ anomeric carbon, nicotinamide (NAM) is first released, and an intermediate enzyme-ADPR complex is formed. In the second step, a base attacks the enzyme-ADPR bond to release the product from enzyme. This base could be a water molecule - in which case a free ADPR is formed, or it could be one of a variety of other cellular base molecule, leading to a base-exchange reaction and to the formation of different products (not presented). Alternatively, a self-attack by adenosine N1 results with cyclization and the release of a cADPR molecule.

Figure S3

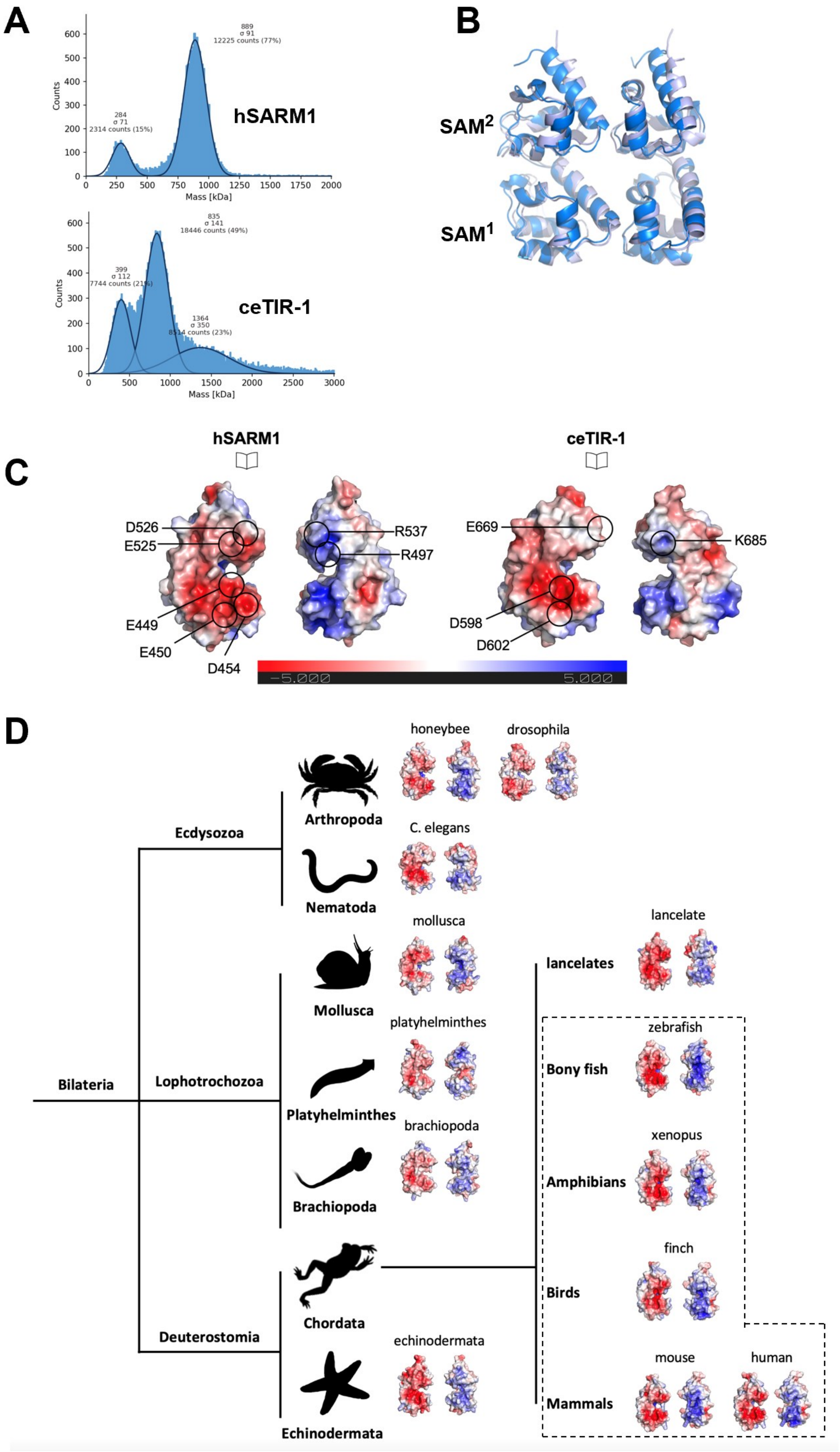
A
B
C
D
hSARM1
ceTIR-1
Counts
Mass [kDa]
889
σ 91
12225 counts (77%)
284
σ 71
2314 counts (15%)
835
σ 141
18446 counts (49%)
399
σ 112
7744 counts (21%)
1364
σ 350
8514 counts (23%)
SAM2
SAM1
D526
E525
E449
E450
D454
R537
R497
E669
D598
D602
K685
-5.000
5.000
honeybee
drosophila
Arthropoda
Ecdysozoa
C. elegans
Nematoda
mollusca
Mollusca
platyhelminthes
Platyhelminthes
Bilateria
Lophotrochozoa
brachiopoda
Brachiopoda
Chordata
Deuterostomia
echinodermata
Echinodermata
lancelate
lancelates
zebrafish
Bony fish
xenopus
Amphibians
finch
Birds
mouse
human
Mammals

Figure S3. Oligomerization and SAM domain analysis of hSARM1 and ceTIR-1.

A) Mass photometry analyses of purified hSARM1 and ceTIR-1 show a more uniform size distribution in hSARM1 than ceTIR-1, further supporting size heterogeneity of the ceTIR-1 sample, with both smaller and larger oligomers. B) Superposition of two neighboring SAM^{1-2} domains from hSARM1 octamer (blue, PDB 7ANW) and ceTIR-1 9-mer (gray, this work). The structures are closely resembling with RMSD of 1.6 Å for 131 residues superimposed.

C) Complementarity in electrostatic potential in the SAM^{1-2} oligomeric interface. ceTIR-1 (right panel) shows a weaker electrostatic attraction force between neighboring SAM^{1-2} domains, compared to hSARM1 (left panel). The electrostatic surface potential is colored in blue (positive) and red (negative) and calculated with pymol. D) SARM SAM domains electrostatic analysis across animal phyla and classes. Note that strong complementary electrostatic surfaces are more distinctive in chordates. The models were generated by AlphaFold [41].

Figure S4

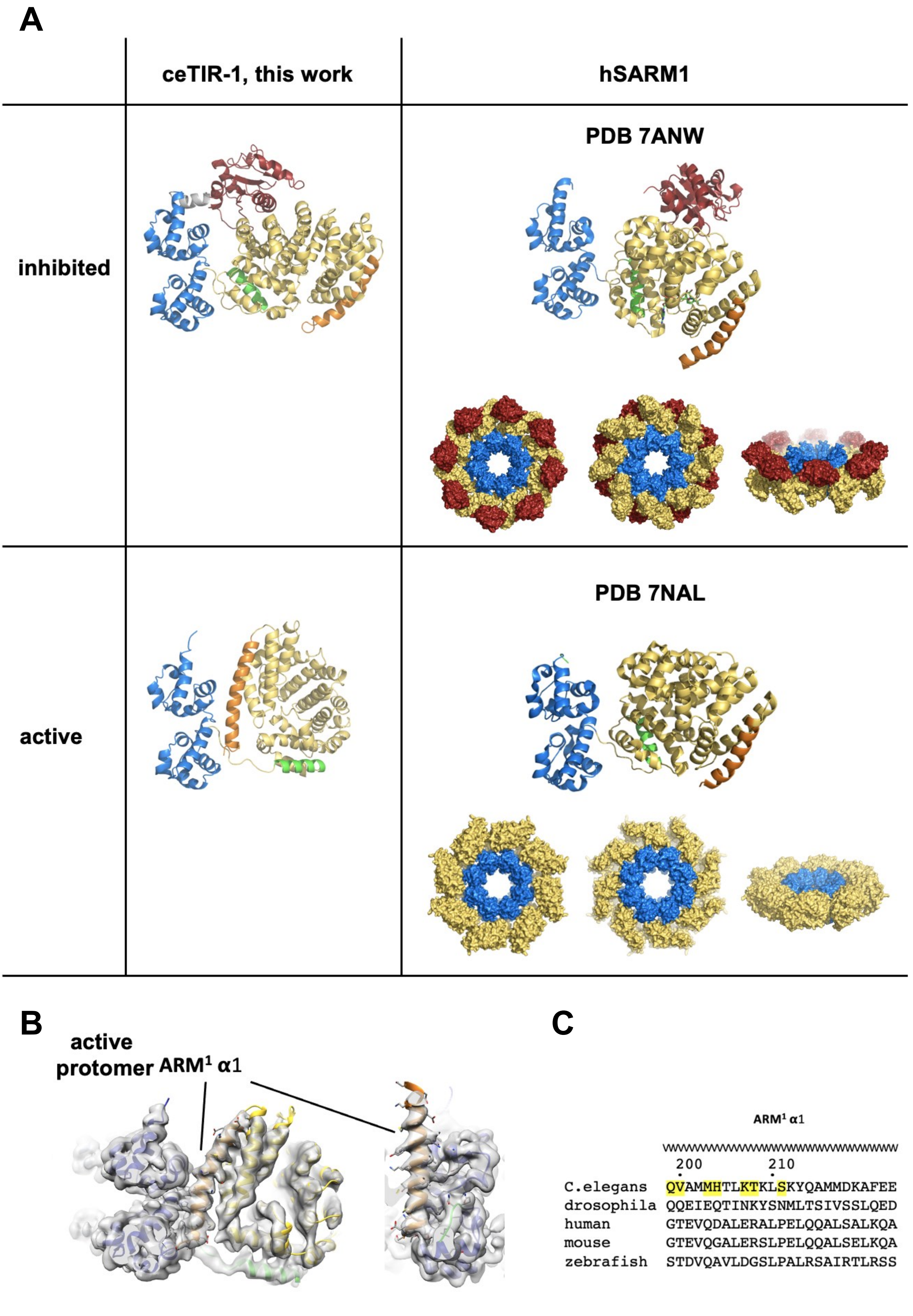

A
ceTIR-1, this work
hSARM1
inhibited
PDB 7ANW
active
PDB 7NAL
B
active
protomer ARM1 α1
C
ARM1 α1
200
210
C.elegans QVAMMHTLKTKLSKYQAMMDKAFEE
drosophila QQEIEQTINKYSNMLTSIVSSLQED
human GTEVQDALERALPELQQALSALKQA
mouse GTEVQGALERSLPELQQALSELKQA
zebrafish STDVQAVLDGSLPALRSAIRTLRSS

Figure S4. Major difference in SAM-ARM interface in active hSARM1/ceTIR-1

A) The positions of the ARM domains in active and inactive states are viewed relative to superimposed SAM^{1-2} domains. For hSARM1, surface representations (top, bottom, and side views) of the entire inhibited and active octamers are also presented. In the inhibited conformations of both ceTIR-1 (a conformation that exists in one of the nine protomers) and hSARM1, the TIR domain (red) is docked onto the ARM domain, where the ARM^2-$\alpha5$ carboxy terminal helix (colored in green) is positioned closest to SAM^{1-2}, and the amino terminal ARM^1-$\alpha1$ (colored in orange) – the furthest away. This conformation is kept also in the active hSARM1 (although with an upward 19 Å translation and a 22° rotation). However, in the other eight active protomers of ceTIR-1, the ARM domains are arranged completely the other way around, with the ARM^1-$\alpha1$ closest to the SAM^{1-2} domains, engaging in several close hydrophobic, polar, and electrostatic interactions with both SAM^1 and SAM^2 (see also in B). C) Sequence alignment of the residues on the ceTIR-1 ARM^1-$\alpha1$ that directly interact with SAM^{1-2} (highlighted in yellow) reveals a complete lack of conservation that makes it unlikely that hSARM1 would adopt such a conformation.

Figure S5

ceTIR-1

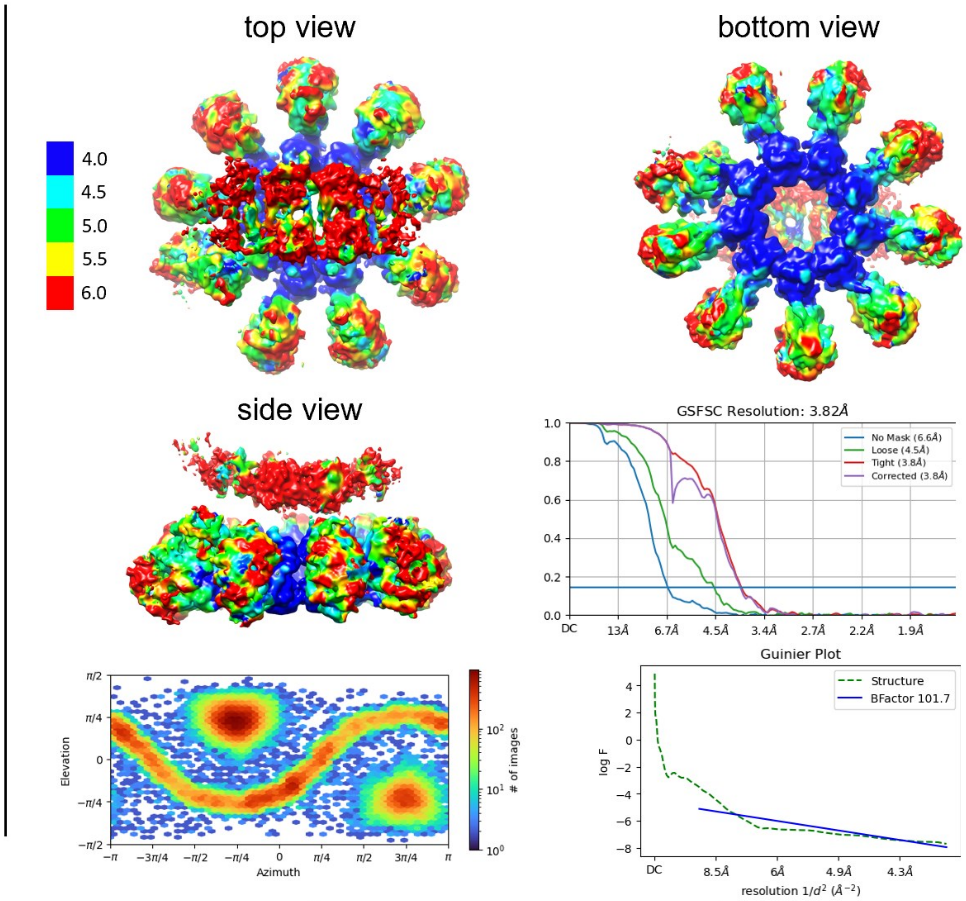

CHIMERA

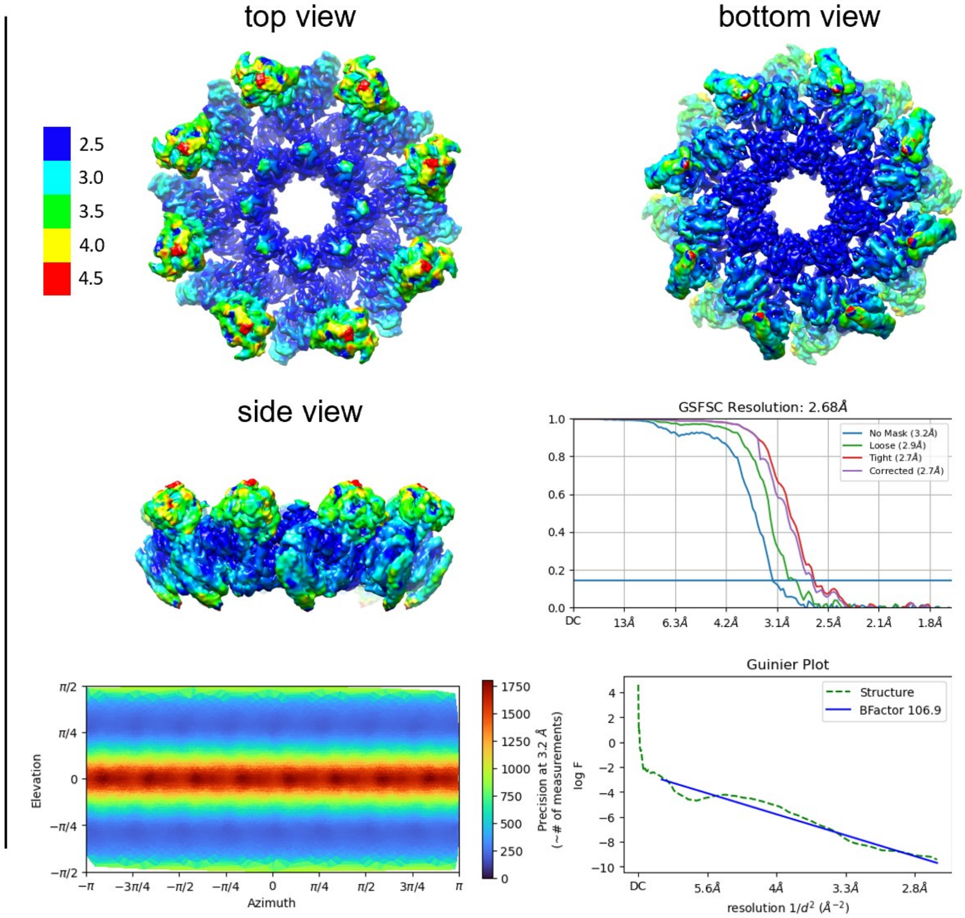

Figure S5. Resolution, angular distribution, and B-factor estimations of the cryo-EM maps of ceTIR-1 and CHIMERA.

Figure S6

A

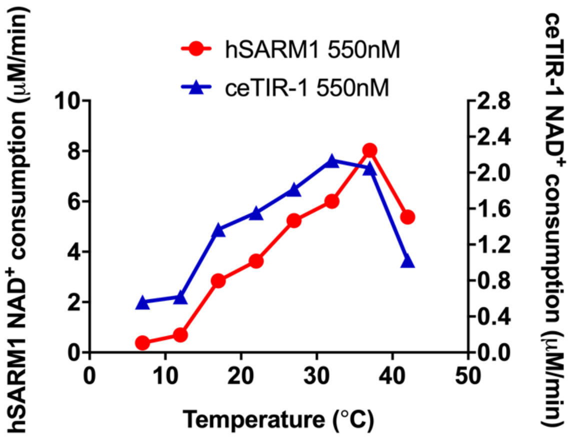

B

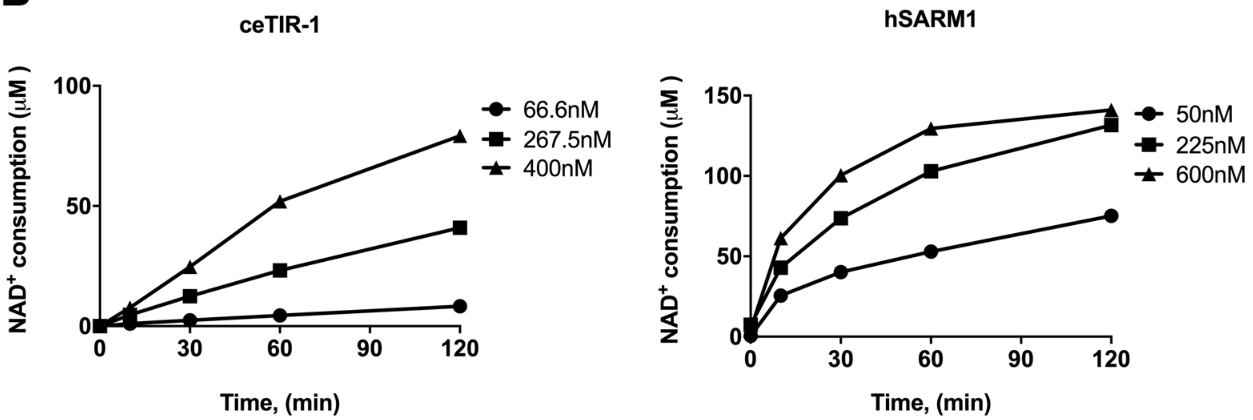

Figure S6. Temperature- and time- dependent activities of ceTIR-1 and hSARM1.A) Temperature dependent activity of ceTIR-1 and hSARM1. Equal concentration of both the proteins (260 nM) were incubated with 400 μM NAD+ for 30 min. At 27°c ceTIR-1 is 88% active compared to 37°c. In hSARM1, the activity in 27°C is 65% of the maximal activity in 37°C. B) Time dependent NADase activity comparison between ceTIR-1 and hSARM1. Different concentrations of ceTIR-1 and hSARM1 were incubated with 150 μM NAD+ for 10, 30, 60 and 120 min at 27°C and 37°C respectively as measured by HPLC. Note that the linear phase of NADase activity in ceTIR-1 is considerably prolonged compared with hSARM1. Data points represent mean of three measurements.

Figure S7

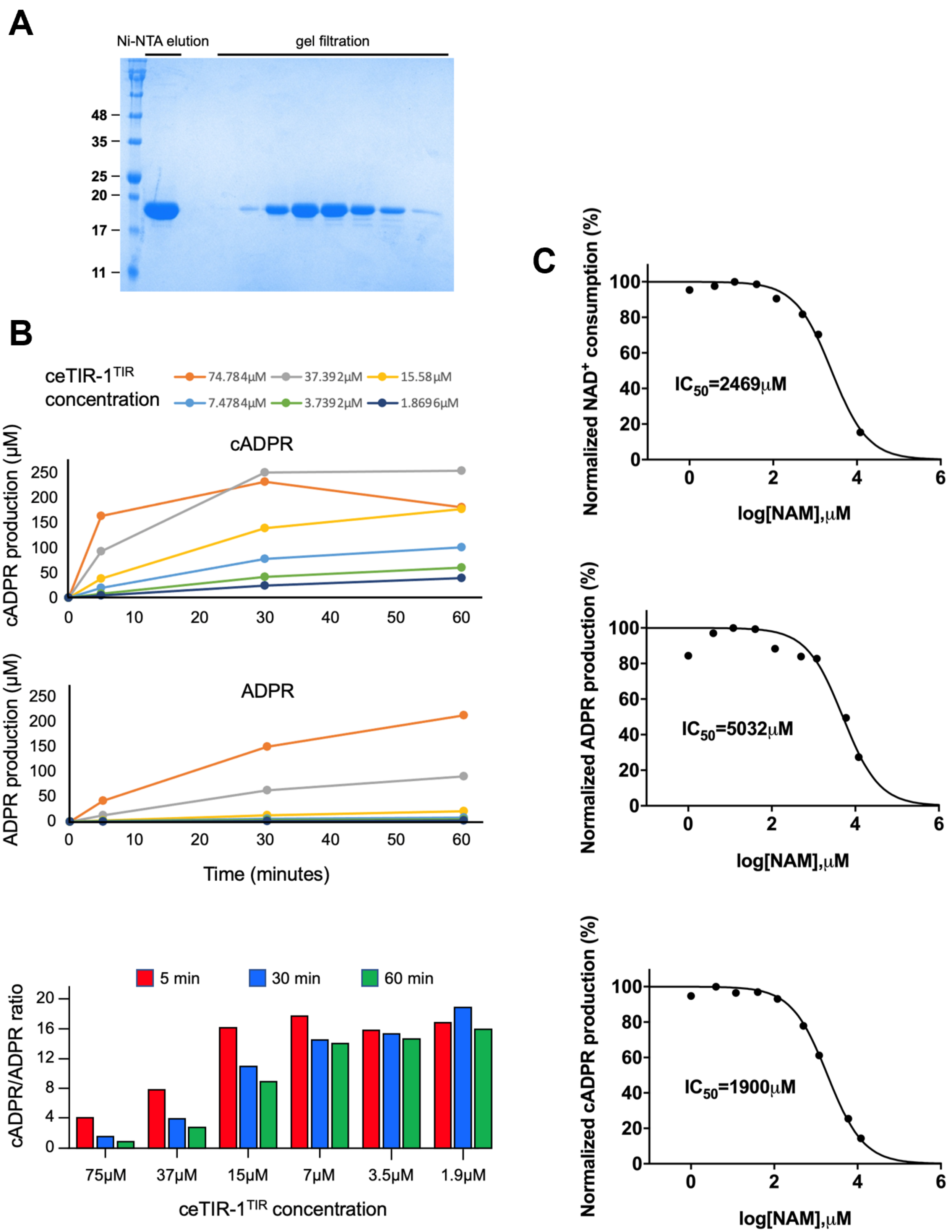
A
Ni-NTA elution
gel filtration
48
35
25
20
17
11
B
ceTIR-1TIR concentration
74.784μM
37.392μM
15.58μM
7.4784μM
3.7392μM
1.8696μM
cADPR
cADPR production (μM)
ADPR
ADPR production (μM)
Time (minutes)
5 min
30 min
60 min
cADPR/ADPR ratio
75μM
37μM
15μM
7μM
3.5μM
1.9μM
ceTIR-1TIR concentration
C
Normalized NAD+ consumption (%)
IC50=2469μM
log[NAM],μM
Normalized ADPR production (%)
IC50=5032μM
log[NAM],μM
Normalized cADPR production (%)
IC50=1900μM
log[NAM],μM

Figure S7. Products of ceTIR-1^{TIR}.

A) Coomassie stained SDS-PAGE of purified ceTIR-1^{TIR}. B) Different concentrations of ceTIR-1^{TIR} were incubated with 500 µM NAD+ for different times at 27°C. Production of cADPR and ADPR and the ratio between them were determined for each ceTIR-1^{TIR} concentration. ceTIR-1^{TIR} concentration increases, less cADPR and more ADPR are produced. C) Determination of IC_{50} values. Various concentrations of NAM were pre-incubated with ceTIR-1^{TIR} for 10 minutes at room temperature, followed by the addition of 300 µM NAD+, initiating the NADase activity at 27°C for 10 minutes. NAM inhibits the production of both ADPR and cADPR.

The amounts of cADPR, ADPR, and NAD+ were measured by HPLC and used to calculate the IC_{50} values by plotting the normalize response to the dose of the NAM (log10). All calculations were performed in GraphPad Prism software.

Figure S8

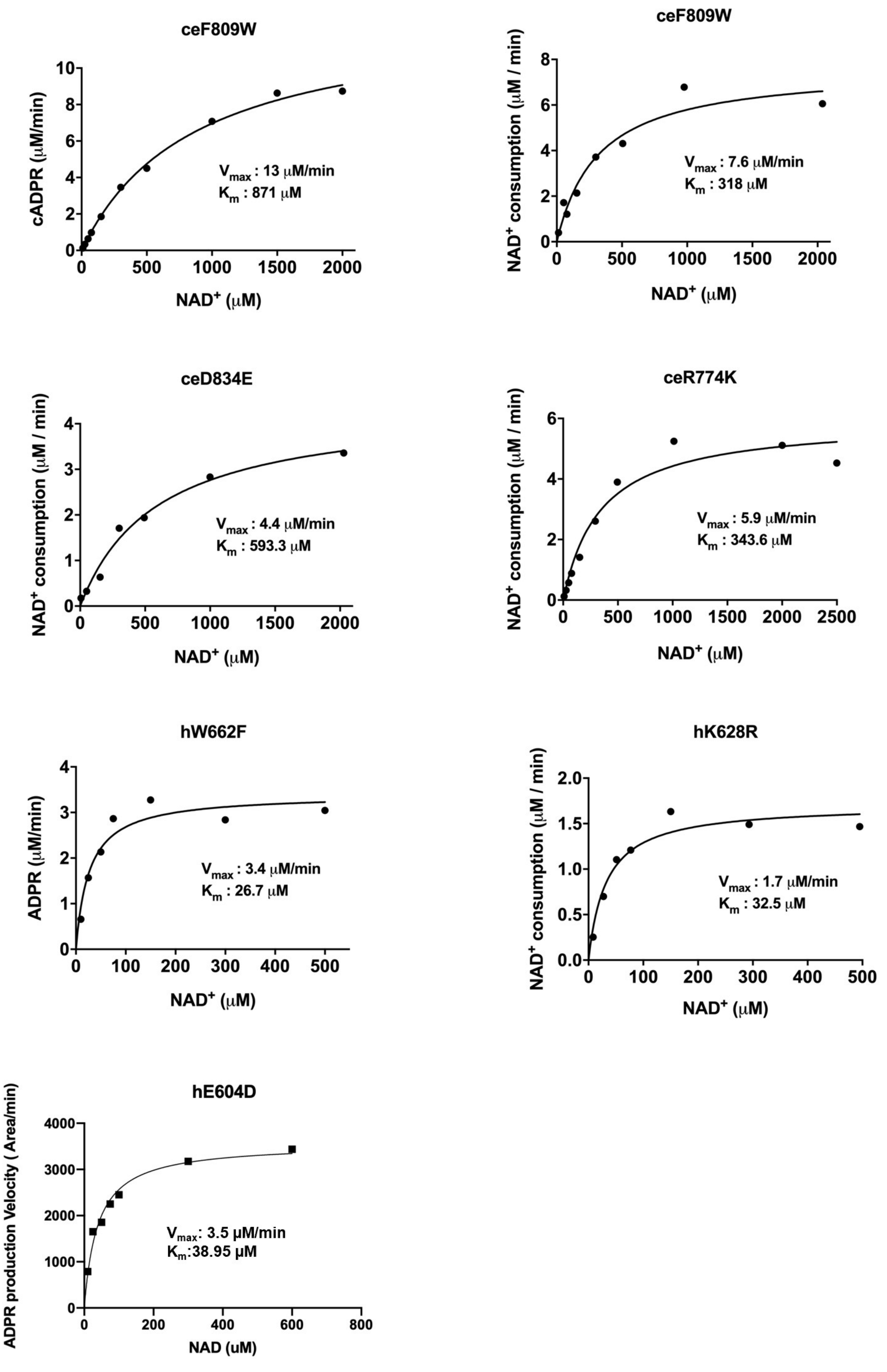
ceF809W
cADPR (μM/min)
NAD+ (μM)
Vmax : 13 μM/min
Km : 871 μM
ceF809W
NAD+ consumption (μM / min)
NAD+ (μM)
Vmax : 7.6 μM/min
Km : 318 μM
ceD834E
NAD+ consumption (μM / min)
NAD+ (μM)
Vmax : 4.4 μM/min
Km : 593.3 μM
ceR774K
NAD+ consumption (μM / min)
NAD+ (μM)
Vmax : 5.9 μM/min
Km : 343.6 μM
hW662F
ADPR (μM/min)
NAD+ (μM)
Vmax : 3.4 μM/min
Km : 26.7 μM
hK628R
NAD+ consumption (μM / min)
NAD+ (μM)
Vmax : 1.7 μM/min
Km : 32.5 μM
hE604D
ADPR production Velocity (Area/min)
NAD (uM)
Vmax: 3.5 μM/min
Km:38.95 μM

Figure S8. Kinetic comparison of different hSARM1 and ceTIR-1 mutants.

The proteins were incubated with varying substrate concentration for 10 minutes (ceTIR-1^{D834E}, ceTIR-1^{R774K}, hSARM1^{E604D} and hSARM1^{W662F}) or for 30 minutes (hSARM1^{K628R} and ceTIR-1^{F809W}).

The kinetic parameters were determined from plots of reaction velocity of NAD+ consumption versus substrate (NAD+) concentration and then fitted to the Michaelis-Menten equation (*K*m and *V*max) using non-linear curve fit in GraphPad Prism.
