# Supplementary material for: Structure-function analysis of ceTIR-1/hSARM1 explains the lack of Wallerian axonal degeneration in C. elegans
Source: Cell Rep. Author manuscript; Available in PMC 2023 Nov 25. (PMC10675840; doi:10.1016/j.celrep.2023.113026)
Supplement: 2 [file NIHMS1933881-supplement-2.docx]

**Table S1**

**Cryo-EM data acquisition, reconstruction and model refinement statistics.**

| **hSARM1 construct and pre-treatment** | | | | | **ceTIR-1** | | | | | **CHIMERA**  **+5mM NAD^+^** | | |
| --- | --- | --- | --- | --- | --- | --- | --- | --- | --- | --- | --- | --- |
| **Electron microscope** | | | | | Titan Krios | | | | | Titan Krios | | |
| **Cryo-EM acquisition and processing** | | | | |  | | | | |  | |  |
| **EMDB accession #** | | | | | 17370 | | | | | 17369 | | |
| **Magnification** | | | | | 105,000x | | | | | 105,000x | | |
| **Voltage (kV)** | | | | | 300 | | | | | 300 | | |
| **Total electron exposure (e^-^ / Å^2^)** | | | | | 41.4 | | | | | 41.4 | | |
| **Defocus range (μM)** | | | | | -0.8 to -2.8 | | | | | -0.8 to -2.8 | | |
| **Pixel size (Å)** | | | | | 0.84 | | | | | 0.84 | | |
| **Symmetry imposed** | | | | | None (C1) | | | | | C8 | | |
| **Initial particles** | | | | | 370,021 | | | | | 298,564 | | |
| **Final particles** | | | | | 121,487 | | | | | 136,346 | | |
| **Resolution (masked FSC = 0.143, Å)** | | | | | 3.82 | | | | | 2.68 | | |
| **Model Refinement** | | | |  | | | | |  |  |  |  |
| **PDB ID** | | | | | 8P2M | | | | | 8P2L | | |
| **Model/map resolution (FSC = 0.50/0.143Å)** | | | | | 4.5 / 3.8 | | | | | 3.3 / 2.7 | | |
| **Model refinement resolution** | | | | | 3.82 | | | | | 2.68 | | |
| **Non-hydrogen atoms** | | | | | 46,727 | | | | | 40,792 | | |
| **Residues** | | | | | 5,819 | | | | | 5,152 | | |
| **RMS deviations** | | |  | | | | |  | | | | |
| **Bond length (Å)** | | | | | 0.010 | | | | | 0.009 | | |
| **Bond angle (°)** | | | | | 1.83 | | | | | 1.64 | | |
| **Ramachandran plot** | |  | | | | |  | | | | | |
| **Favored (%)** | | | | | 95.6 | | | | | 97.2 | | |
| **Allowed (%)** | | | | | 4.4 | | | | | 2.8 | | |
| **Disallowed (%)** | | | | | 0.0 | | | | | 0.0 | | |
| **Rotamer Outliers (%)** | | | | | 1.90 | | | | | 0.56 | | |
| **Validation** |  | | | | |  | | | | |  |  |
| **MolProbity score** | | | | | 1.54 | | | | | 0.96 | | |
| **Clashscore** | | | | | 2.42 | | | | | 1.07 | | |
