# Supplementary material for: Structure-function analysis of ceTIR-1/hSARM1 explains the lack of Wallerian axonal degeneration in C. elegans
Source: Cell Rep. Author manuscript; Available in PMC 2023 Nov 25. (PMC10675840; doi:10.1016/j.celrep.2023.113026)
Supplement: 3 [file NIHMS1933881-supplement-3.docx]

**Table S2**

*C. elegans* strains

| Strain | Allele | Plasmids | Description |
| --- | --- | --- | --- |
| XE3145 | ***wpEx531***[P*itr-1*::humanSARM1::SL2::mCherry-H2B:: *let-858* UTR, P*mig-13*::myr-EGFP::*let-858* UTR, *Pmyo-2*::mCherry::*unc-54* UTR] | **pHT032**  P*mig-*13::myristoylated-EGFP:: *let-858*-UTR  in pDest r4-r3    Made by Gateway  Injected at 15ng/μL    **pHT026**  P*itr-*1::humanSARM1::SL2:: mCherry-H2B::*let-858*-UTR  in pDest r4-r3    Made by Gateway  Injected at 25ng/μL    **pCFJ90**  P*myo-*2::mCherry::*unc-54* UTR | Expressed in DA9: Codon optimized human SARM1 with generic introns added, followed by an SL2 sequence and histone-localized mCherry (used for expression level quantification). Membrane localized mEGFP as cell-shape marker.  Pharyngeal mCherry as co-injection marker. |
| XE3146 | **wpEx532**[P*itr-1*::TIR-1::sl2::mCherry-H2B:: *let-858* UTR, P*mig-13*::myr-EGFP::*let-858* UTR, *Pmyo-2*::mCherry::*unc-54* UTR] | **pHT032**  P*mig-*13::myristoylated-EGFP:: *let-858*-UTR  in pDest r4-r3    Made by Gateway  Injected at 15ng/μL    **pHT033**  P*itr-1*::TIR-1::sl2::mCherry-H2B:: *let-858* UTR in pDest r4-r3    Made by Gateway  Injected at 25ng/μL    **pCFJ90**  P*myo-*2::mCherry::*unc-54* UTR | Expressed in DA9: *C.elegans* TIR-1 with generic intron added followed by an SL2 sequence and histone-localized mCherry (used for expression level quantification). Membrane localized mEGFP as cell-shape marker.  Pharyngeal mCherry as co-injection marker. |
