# Supplementary material for: Structure-function analysis of ceTIR-1/hSARM1 explains the lack of Wallerian axonal degeneration in C. elegans
Source: Cell Rep. Author manuscript; Available in PMC 2023 Nov 25. (PMC10675840; doi:10.1016/j.celrep.2023.113026)
Supplement: 4 [file NIHMS1933881-supplement-4.docx]

**Table S3**

Primer sequences

| **Primer name** | **Sequence** | **Description** |
| --- | --- | --- |
| YW462 | ATGGGACCAACCTAACACCCAGCTTTCTTGTACAAAG | forward primer to amplify [1, 2] for Gibson assembly with hSARM1 |
| YW463 | GAGGGTGAGGACCATGAAGCCTGCTTTTTTGTACAAAG | reverse primer to amplify [1, 2] for Gibson assembly with hSARM1 |
| CF15 | taaccctgattatttaaattttcagAACTGACGACGCATTCTTCG | forward primer for generic intron insertion into ceTIR-1 |
| CF16 | gttagtatatatatgtttaaacttacCGTCCAAGTGGACTGCGG | reverse primer for generic intron insertion into ceTIR-1 |
